# MEF2C controls lysosomal and lipid clearance programs linked to Alzheimer’s disease risk in macrophages

**DOI:** 10.21203/rs.3.rs-9164252/v1

**Published:** 2026-04-28

**Authors:** Anna Podlesny-Drabiniok, Jeanne J. Kim, Lotte J. Bezemer, Tulsi Patel, Haoxiang Cheng, Michael Sewell, Mia Montecillo, Nicholas Church, Anthony Walley, Francesca Garretti, Edoardo Marcora, Alison M. Goate

**Affiliations:** 1 Ronald M. Loeb Center for Alzheimer’s Disease, Department of Genetics & Genomic Sciences, Icahn School of Medicine at Mount Sinai, 1 Gustave L. Levy Place, New York, NY 10029, USA.; 2 Sanford Grossman Interdisciplinary Program In Neural Circuitry and Immune Function; 3 Present address: Center for Neurogenomics and Cognitive Research, Vrije Universiteit Amsterdam, Amsterdam Neuroscience, Amsterdam, the Netherlands; 4 Present address: Alzheimer Center Amsterdam, Department of Neurology, Amsterdam University Medical Center, Amsterdam Neuroscience, Amsterdam, the Netherlands; 5 Marc and Jennifer Lipschultz Precision Immunology Institute, Icahn School of Medicine at Mount Sinai, New York, NY, USA; 6 Graduate School of Biomedical Sciences, Icahn School of Medicine at Mount Sinai, New York, NY, USA

## Abstract

Risk alleles for late-onset Alzheimer’s disease (AD) are enriched in myeloid cis-regulatory elements, implicating myeloid gene-regulatory networks in disease susceptibility. A conserved lipid-associated transcriptional signature—spanning disease-associated microglia and peripheral lipid-associated macrophages (DLAM)—emerges across neurodegenerative and metabolic diseases characterized by lipid overload, yet the transcriptional regulators of this gene expression program remain incompletely defined. Here, we show that MEF2C—a candidate AD risk gene—is a master DLAM regulator. Using MEF2C knockout and knockdown in human iPSC-derived microglia and macrophages, we found that total or partial MEF2C loss is sufficient to induce DLAM-associated transcriptional, epigenomic, and functional remodeling, including enhanced lysosomal activity and cholesterol efflux. Integration of chromatin accessibility and regulatory epigenetic profiles with functionally informed fine-mapping linked candidate causal variants in AD risk loci to MEF2C-regulated cis-regulatory elements that target candidate AD risk genes at these loci. In a triculture model of AD, microglial MEF2C loss is associated with an increased DLAM population and a reduced Aβ42/40 ratio, supporting context-dependent reprogramming of microglia as a potential biological mechanism to modulate AD-relevant pathology.

## Introduction

Alzheimer’s disease (AD) is a progressive and ultimately fatal neurodegenerative disorder with a substantial genetic component encompassing several AD risk genes^1^. It is the leading cause of dementia worldwide, accounting for 60–80% of cases in the elderly^2^. Despite decades of basic and clinical research, no effective disease-modifying therapies exist, and the biological mechanisms through which genetic variation confers risk or protection remain incompletely understood.

Human genetic studies have transformed our understanding of the molecular and cellular basis of the common, late-onset, sporadic, and polygenic form of AD. Both common and rare variants converge on pathways central to efferocytosis and innate immunity^3,4^. Genome-wide association studies (GWAS) have identified numerous non-coding cis-regulatory variants associated with AD risk, with AD-associated alleles showing striking enrichment in enhancers active in myeloid cells—including microglia, the resident macrophages and innate immune effectors of the CNS^5–7^. These observations highlight microglial and macrophage processes as key determinants of disease susceptibility, with cholesterol metabolism, phagocytic clearance, and inflammatory signaling repeatedly emerging as central pathogenic processes^8–10^.

Among microglial transcriptional signatures described across human and mouse AD models, one of the most reproducible is the disease-associated microglia (DAM) signature^11–16^. DAM is enriched for efferocytosis-related pathways, including phagocytic, lysosomal, lipid-handling, and immune response pathways—precisely the processes implicated by AD genetics^4,10^. We and others have shown that DAM share substantial transcriptional and functional similarities with lipid-associated macrophages (LAM) in adipose tissue^17^ and with foamy macrophages surrounding atherosclerotic plaques^18,19^, including upregulation of genes involved in lipid and cholesterol processing such as *APOE*, *CD68*, *TREM2*, and *ABCA1*. We collectively refer to these brain-resident and peripheral macrophage subsets as DLAM, reflecting their shared molecular signatures^18,20^. Previously, we reconstructed DLAM-specific gene regulatory networks (GRNs) and nominated a set of transcription factors (TFs) that orchestrate DLAM responses. Among these we found several TFs that map to AD risk loci including *SPI1*, *BHLHE40*, *BHLHE41*. Another strong candidate that emerged from this study was myocyte enhancer factor 2C (MEF2C)^20^. Importantly, *MEF2C* has been nominated as the candidate target gene at *MEF2C*-labelled AD GWAS risk locus^21^, linking MEF2C directly to genetic susceptibility.

In the CNS, MEF2C is expressed prominently in microglia as well as in excitatory and inhibitory neurons; however the biological functions of MEF2C differ by cell type. In neurons, MEF2C regulates synaptic development and cognitive function: reduced neuronal MEF2C impairs synaptic plasticity–related transcriptional programs and contributes to cognitive decline, whereas higher MEF2C expression is associated with cognitive resilience^22,23^. Neuronal MEF2C target genes are enriched for heritability of schizophrenia, IQ, and educational attainment^24^. Furthermore, MEF2C loss-of-function mutations cause MEF2C haploinsufficiency syndrome (MCHS), a severe neurodevelopmental disorder with autism-spectrum features^25^. Children with MCHS have lower levels of MEF2C in the hippocampus that contributes to reduced neurogenesis^26^.

In contrast, the role of microglial MEF2C is far less well characterized. Genome-wide CRISPR interference screens have strongly implicated MEF2C as an essential regulator of microglial and other myeloid lineages^27^. MEF2C has also been proposed as a key regulator of chromatin accessibility in brain-resident macrophages^14^. Consistent with this role, MEF2C is necessary to establish high CX3CR1 expression in human and murine microglia^28,29^. Existing studies show that microglial MEF2C expression declines with aging and is reduced following inflammatory stimulation^29^. Loss of MEF2C in microglia leads to exaggerated induction of pro-inflammatory cytokines—including IL-1β, TNFα, and IL-6—in response to LPS or TNFα, consistent with MEF2C acting as an immune checkpoint that suppresses NF-κB nuclear translocation and prevents excessive microglial activation, neuronal injury, and autism-like behaviors^29–31^. Microglial MEF2C target genes were recently shown to be enriched for bipolar disorder and autism heritability^31^, further implicating MEF2C in neuroimmune–neuropsychiatric cross-talk. Functionally, MEF2C-deficient microglia exhibit altered lysosomal content, impaired migration, and reduced phagocytosis^31^, although conflicting data report increased phagocytosis for specific substrates^30,31^, suggesting substrate- or context-specific MEF2C-dependent regulation.

Although myeloid cells represent a genetically supported causal cell type in AD, and MEF2C has been implicated as an important regulator of microglial biology, the function of microglial MEF2C specifically in the context of AD remains poorly understood. Clinical data report reduced MEF2C mRNA levels in leukocytes from Japanese AD patients^32^. One study reported that anti-IFNAR blockade alters microglial cytoplasmic-to-nuclear MEF2C translocation^33^, yet the functional consequences of this translocation remain unexplored. This underscores a critical knowledge gap: how MEF2C, as an important microglial transcription factor and putative AD risk gene, shapes microglial responses relevant to AD pathogenesis. Comprehensive characterization of microglial MEF2C function in AD-relevant environmental and genetic contexts is therefore essential to understand how MEF2C contributes to AD risk and resilience.

## Results

### Functional MEF2C targets are enriched across myeloid cluster marker genes, AD risk genes, and age-associated microglial transcriptomic changes

We have previously nominated MEF2C as a key regulator of DLAM responses in at least half of the gene regulatory networks generated in both mouse and human^20^. Using proxy-binding sites defined by open chromatin regions and the presence of MEF2C motifs, we estimated that MEF2C binds approximately 18% of DLAM genes, particularly lipid-associated macrophage genes derived from adipose tissue^20^. Here, we refine this analysis using experimentally validated binding sites from recently published microglial MEF2C ChIP-seq data^31^. We reanalyzed the data (Methods, Supplementary Data 1) and restricted the analysis to MEF2C ChIP-seq peaks located at gene promoters, thereby prioritizing binding events with the highest likelihood of direct transcriptional regulation. To identify direct functional targets of MEF2C in microglia, we focused on the intersection between MEF2C ChIP-seq promoter-bound targets (6,824 genes) and MEF2C regulons (6,461 genes) (Supplementary Data 1). This analysis yielded 2,955 functional MEF2C targets, with a highly significant hypergeometric overlap between MEF2C direct targets and MEF2C regulon genes (p = 5.18 × 10e-122, one-sided) (Supplementary Data 1).

**Figure 1.**
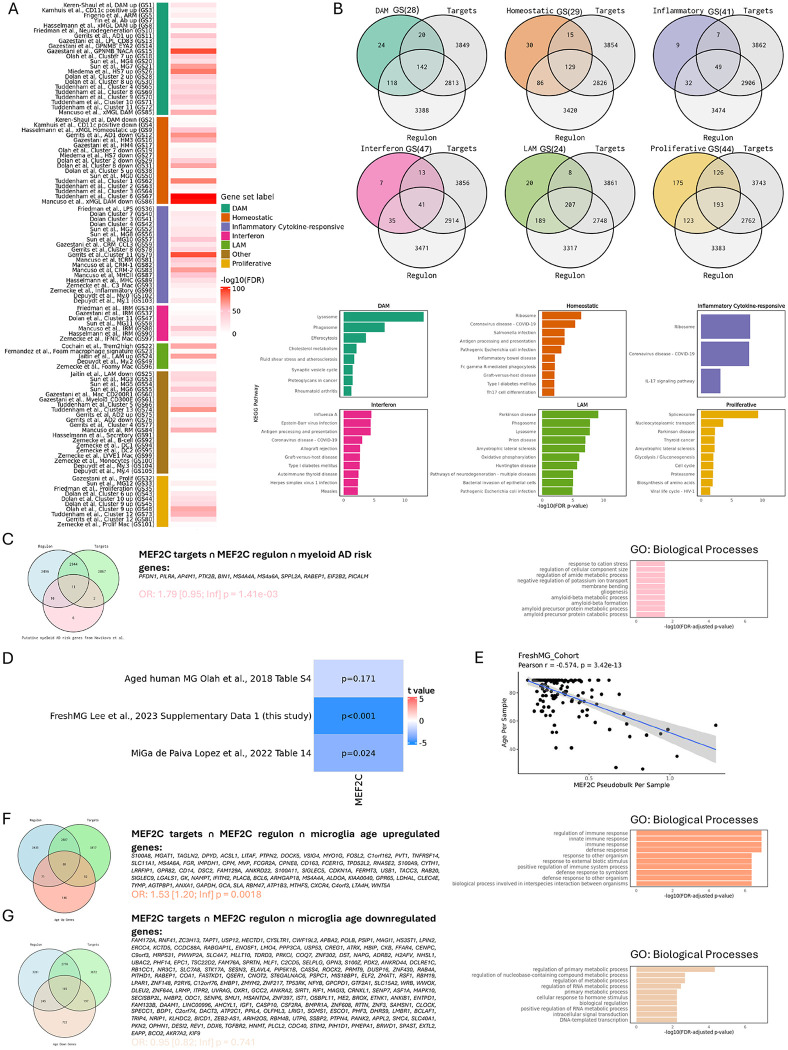
Functional myeloid MEF2C targets are enriched in age- and disease-associated transcriptional programs **(A)** Enrichment of published myeloid gene sets in functional myeloid MEF2C targets, displayed as −log10(FDR-corrected p-value of overlap). Gene sets associated with disease- and lipid-associated microglia (DAM/LAM), inflammatory, interferon-responsive, homeostatic, and proliferative states are listed in Supplementary Data 1. **(B)** Overlap between curated myeloid gene sets, MEF2C regulon and MEF2C binding targets. Venn diagrams depict shared and unique genes between each myeloid gene set, MEF2C regulon genes, and targets bound at the promoter, with pathway enrichment analyses shown below for overlapping genes. Curated myeloid gene sets derived from *Dolan et al*.^43^ For details see Supplementary Data 1 (this study) **(C)** Intersection of MEF2C binding targets and MEF2C regulon genes with putative myeloid AD risk genes nominated by Novikova *et al*^7^. Pathway enrichment analysis of shared genes. **(D)** MEF2C expression across aged human microglia from three independent transcriptomic datasets from^44,84^ across the lifespan. Each row indicates MEF2C expression (t value) derived from the original study, with statistical significance assessed as indicated (p indicated adjusted p value). **(E)** The scatter plot illustrates the inverse correlation between MEF2C expression and age in human microglial samples. FreshMG was reanalyzed (see Methods) and the results are listed in Supplementary Data 1. **(F,G)** Overlap between MEF2C binding targets and regulon genes with microglial genes upregulated (**F**) or downregulated (**G**) with aging from^44^. Pathway analysis of age-upregulated and age-downregulated intersections. In C, F, G one-side Fisher’s exact test was used, odds ratio, 95% confidence interval and p-value are reported in the figure. All input lists and pathway analysis are listed in Supplementary Data 1.

We next assessed the enrichment of these functional MEF2C targets across more than one hundred microglial and peripheral macrophage transcriptional signatures derived from amyloidosis^15,34–37^, demyelination^38^, atherosclerosis^18,19,39,40^, and obesity models^17^, and Alzheimer’s disease (AD) brains^11–13,16,41^ referred to as myeloid gene sets (Supplementary Data 1). Those RNAseq signatures represent major microglial transcriptional states identified in sc/snRNAseq studies and include disease-associated microglia (DAM), homeostatic, inflammatory/cytokine-responsive, interferon, proliferative, lipid-associated macrophage (LAM) states. MEF2C functional targets were not restricted to a single state but exhibited significant enrichment across multiple gene sets (Figure 1A). The most pronounced overlaps were observed with marker genes of the DAM cluster from the human brain GPNMB_NACA dataset^12^, the MG7 cluster enriched in glycolysis^11,12^, and two homeostatic clusters, including human Cluster 6^13^ and xMGL from chimeric mouse models^42^ (xMGL DAM downregulated). FDR-corrected p-values for all myeloid gene set overlaps are listed in Supplementary Data 1.

To better understand the biological processes regulated by MEF2C in distinct myeloid states, we selected representative signatures^43^ for each state and performed pathway analysis on genes intersecting with functional MEF2C targets. In DAM and LAM states, functional MEF2C targets are enriched for lysosomal and phagosomal pathways, efferocytosis, and cholesterol metabolism; in homeostatic microglia, they are enriched for ribosomal function as well as antigen processing and presentation. In inflammatory or cytokine-responsive myeloid states, functional MEF2C targets were enriched for ribosomal and IL-17 signaling pathways, while in proliferative states, functional MEF2C targets are enriched for spliceosome, cell cycle, and proteasome pathways (Figure 1B). Complete pathway identifiers and FDR-corrected p-values are reported in Supplementary Data 1.

Given that MEF2C was proposed as a candidate gene in the first AD GWAS at the MEF2C locus^21^, we assessed its potential role in AD by intersecting functional MEF2C targets with genes^7^ that we previously nominated as candidate AD risk genes in myeloid cells (Fig.5 in^7^ and Supplementary Data 1). We observed a significant positive overlap (OR = 1.79 [0.95, Inf], Fisher’s exact test p = 1.41 × 10e-3, two-sided) encompassing 11 myeloid AD risk genes enriched among MEF2C functional targets. These genes are involved in amyloid-beta metabolic process, gliogenesis and regulation of amide metabolic process (Figure 1C).

Genetic association of MEF2C with AD risk highlights this TF as a potential regulator of microglial pathways that contribute to AD risk. This is particularly relevant in the context of aging, where MEF2C is downregulated across three independent human microglial datasets with age (Figure 1D). Consistently, we identified a significant inverse correlation between MEF2C expression and age (ρ = −0.574, p=3.42×10^−13^) (Figure 1E). This observation prompted an analysis of functional target overlap with age-associated genes from the human microglia dataset^44^. We found significant enrichment among genes upregulated with age (OR = 1.53 [1.20, Inf], Fisher’s exact test p = 0.0018, two-sided), which were involved mainly in immune response biological processes (Figure 1F). In contrast, genes downregulated with age showed no statistically significant overlap with functional MEF2C targets (OR = 0.95 [0.82, Inf], Fisher’s exact test p = 0.741). GO Biological Process analysis revealed enrichment in regulation of metabolic processes. Together, these data suggest that functional MEF2C targets may operate across diverse myeloid states, contribute to AD polygenic risk, and are affected by aging.

### Loss of MEF2C in iPSC-derived microglia leads to upregulation of DLAM transcriptional programs

**Figure 2.**
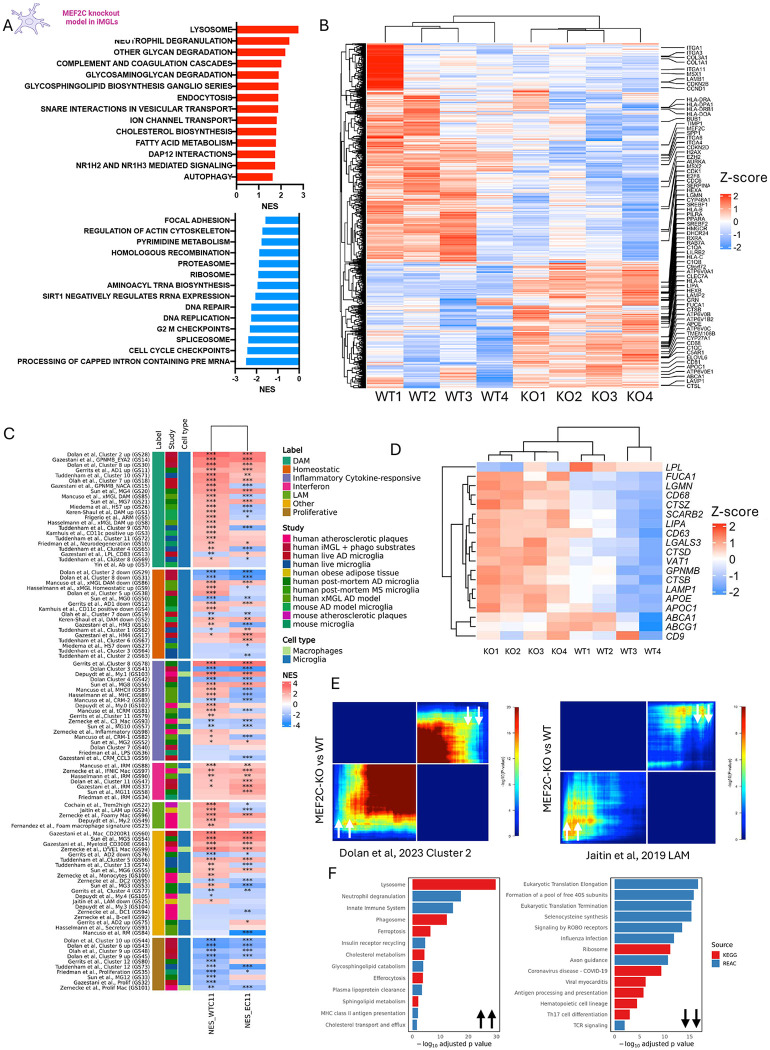
MEF2C loss increases lysosomal and lipid metabolism genes and induces DLAM myeloid transcriptional responses **(A)** Gene set enrichment analysis of MEF2C-KO transcriptome in iMGLs relative to WT controls. Bar plots show the top positively (red) and negatively (blue) enriched pathways ranked by normalized enrichment score (NES) **(B)** Heatmap of z-scored expression levels for genes contributing to the top enriched pathways in KO versus WT iMGLs. **(C)** Enrichment of published myeloid gene signatures across two MEF2C-KO iMGL lines WTC11 (this study), and EC11^31^. Heatmap shows normalized enrichment scores (NES) for myeloid gene sets derived from human and mouse studies (Supplementary Data 1), annotated by study source and cell type. Details in Supplementary Data 2 **(D)** Z-scored expression heatmap of canonical DLAM marker genes across KO and WT iMGLs, demonstrating coordinated upregulation of lipid handling, lysosomal, and phagocytic genes upon MEF2C loss. **(E)** Rank–rank hypergeometric overlap (RRHO) analysis comparing the MEF2C-KO transcriptional signature (KO_NT vs WT_NT) with disease-associated microglia Cluster 2(from^43^ Supplementary Table 2, Cluster 2) and lipid-associated macrophages from (^17,43^, Dataset S6, FDR < 0.05). **(F)** Functional enrichment analysis of genes contributing to the most significant RRHO overlap regions. Bar plots show enriched KEGG and Reactome pathways. For pathway analysis, concordantly upregulated genes (lower-left quadrants) and concordantly downregulated genes (upper-right quadrants) from DAM and LAM RRHO comparisons with the MEF2C-KO transcriptome were combined. Input gene lists and corresponding pathway enrichment results for panels E and F are provided in Supplementary Data 2.

To further characterize the role of MEF2C in myeloid cells, and understand the effects of MEF2C downregulation, we utilized CRISPR–Cas9-mediated insertion of a STOP codon in exon 3 to disrupt MEF2C expression and generated four independent knockout (KO) clones along with their corresponding wild-type (WT) controls in the WTC11 iPSC line. Following microglial differentiation, loss of MEF2C protein was confirmed by western blotting (Supplementary Figure 1C). To assess the global transcriptomic changes associated with loss of MEF2C, we performed bulk RNAseq of WT and KO iMGLs. Analysis revealed 1,273 upregulated and 904 downregulated genes (FDR < 0.05) (Supplementary Data 2). Upregulated genes were enriched in pathways related to lysosomal degradation (lysosome (map04142), neutrophil degranulation (R-HSA-6798695), endocytosis (hsa04144), autophagy (R-HSA-9612973)), lipid metabolism (glycosphingolipid biosynthesis (R-HSA-1660662), fatty acid metabolism (R-HSA-8978868), NR1H2/3-mediated signaling (R-HSA-9024446), cholesterol biosynthesis (R-HSA-191273)), and synaptic transmission (Ion channel transport (R-HSA-983712), SNARE interactions in vesicular trafficking (map04130)) (Figure 2A). Conversely, downregulated genes were enriched in pathways related to adhesion (focal adhesion (hsa04510), regulation of actin cytoskeleton (hsa04810)), cell cycle (DNA replication (R-HSA-69306), G2/M checkpoint (R-HSA-69481)), and translation (Spliceosome (hsa03040), ribosome (hsa03008), aminoacyl-tRNA biosynthesis (map00970)) (Figure 2A–B, Supplementary Data 1).

Although MEF2C functional targets were enriched across diverse myeloid states, these analyses did not establish the directionality of regulation. To define the direction of MEF2C-dependent regulation and validate our previous bioinformatic predictions that MEF2C functions as a DLAM regulator^20^, we performed gene set enrichment analyses (GSEA) using myeloid gene sets (Supplementary Data 1). The KO transcriptome showed strong positive enrichment of nearly all DAM and LAM gene sets, along with cytokine and interferon-responsive signatures, and concurrent negative enrichment of homeostatic and proliferative gene sets (Figure 2C, Supplementary Data 2). Several core DLAM genes—including *APOE, GPNMB, CTSB, LGALS3*, and *LIPA*—were upregulated in KO iMGLs relative to WT controls (Figure 2D). Interestingly, one homeostatic signature, HM_3^12^, also showed significant positive enrichment in the KO transcriptome. To investigate this unexpected result, we assessed pairwise similarities among all myeloid signatures using the Jaccard index (Supplementary Figure 1A–B). This analysis revealed that HM_3 clusters closely with DAM and LAM gene sets, indicating that this signature captures a partial DLAM transcriptional program. This overlap likely explains its statistically significant positive enrichment in the KO condition, despite its original classification as homeostatic.

To confirm these findings in a second iPSC donor, we leveraged the recently published bulk RNAseq dataset from MEF2C-KO iMGLs derived from the EC11 iPSC line^12,31^. We reprocessed these data using our analysis pipeline (Methods; Supplementary Data 2) and observed moderate concordance between EC11- and WTC11-derived iMGLs at both the transcriptomic level (Spearman’s ρ = 0.53, p < 0.001) and the pathway level (Spearman’s ρ = 0.49, p = 6.91 × 10^−189^) (Supplementary Figure 6C). Rank–rank hypergeometric overlap (RRHO) analysis further revealed a strong positive correlation between EC11- and WTC11-derived iMGLs (Supplementary Figure 6C), indicating that loss of MEF2C in two independent iPSC lines results in similar transcriptional profiles. We next performed GSEA using myeloid gene sets and observed a pattern of enrichment similar to that seen in WTC11-derived iMGLs. Specifically, we detected negative enrichment of proliferative and homeostatic signatures, positive enrichment of interferon-responsive signatures, and enrichment of nearly 60% of DAM gene sets. Notably, the exceptions were humanized signatures derived from mouse studies, which were either not enriched or negatively enriched in EC11 iMGLs following MEF2C inactivation (Figure 2C, Supplementary Data 2).

To further validate the GSEA results, we performed RRHO analysis to more precisely identify statistically significant overlaps between transcriptional changes associated with MEF2C loss in iMGLs and DAM and LAM transcriptional signatures representing microglial and peripheral macrophage states responding to lipid-rich stimuli. This analysis revealed that KO iMGL transcriptomes are highly concordant with the transcriptomic signature of a DAM cluster (cluster 2) derived from iMGLs treated with lipid-rich brain phagocytic substrates *in vitro*^43^. Similarly, we observed significant overlap between KO transcriptomes and LAM signatures from adipose tissue macrophages^17^; notably, the most significant overlaps were concentrated among the top upregulated and downregulated genes, as indicated by warmer colors corresponding to lower adjusted p-values in the lower-left (top upregulated genes) and upper-right (top downregulated genes) quadrants of the RRHO map (Figure 2E). Finally, we extracted DAM- and LAM-associated up- and downregulated genes overlapping with KO transcriptome and performed pathway enrichment analysis (Supplementary Data 2). Overlapping upregulated genes were enriched in pathways related to lysosome, phagosome, efferocytosis, plasma lipoprotein clearance, and cholesterol transport and efflux, whereas overlapping downregulated genes were enriched in pathways involved in eukaryotic translation elongation and termination, ribosome biogenesis, and antigen processing and presentation (Figure 2E, Supplementary Data 2). Of note, RRHO analysis revealed that age-associated human microglial transcriptomic changes closely resemble the MEF2C-KO transcriptome (Supplementary Figure 1E; Supplementary Data 2), with strong concordance among the most up- and downregulated genes. Altogether, these data demonstrate that MEF2C loss induces DLAM transcriptional responses characterized by enhanced lysosomal and lipid-handling programs and suppression of homeostatic and proliferative pathways and that they closely mimic age-associated human microglial transcriptomic changes.

### Loss of MEF2C in iPSC-derived microglia leads to increased proportion of iMGL in DLAM cluster

**Figure 3.**
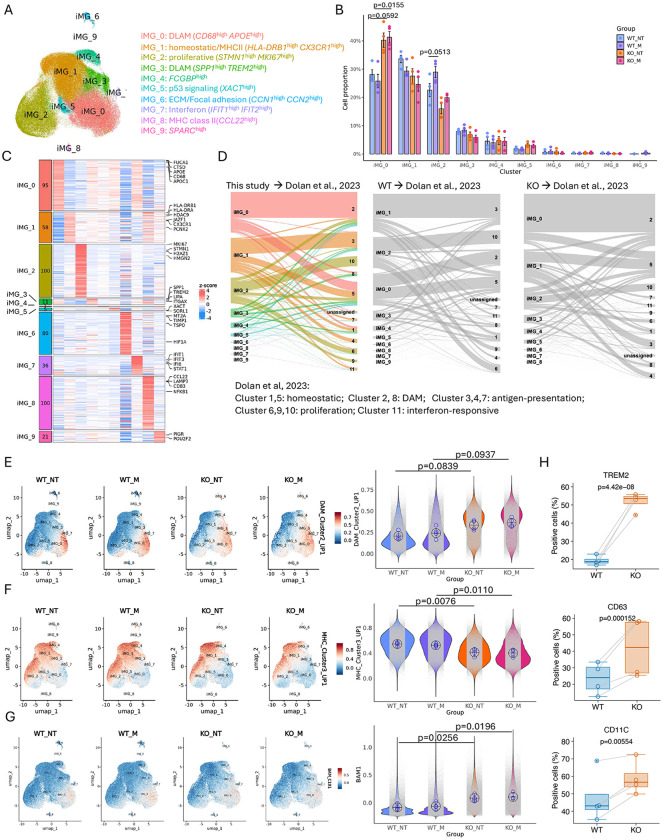
MEF2C loss increases abundance of DLAM clusters and enhances expression of BAM markers. **(A)** UMAP representation of integrated iMGLs identifying ten transcriptionally distinct clusters (iMG_0–iMG_9). Clusters are annotated based on dominant gene signatures, pathway enrichment, and overlap with myeloid gene set (Supplementary Figure 2) **(B)** Quantification of cluster proportions across experimental conditions of KO and WT iMGLs treated with myelin (M) or untreated (NT) (WT_NT, WT_M, KO_NT, KO_M). Bar plots show mean ± s.e.m., with individual clones overlaid (n=4/group). Statistical comparisons highlight genotype- and treatment-dependent shifts in cluster abundance. **(C)** Heatmap of scaled expression (z-scores) for representative marker genes defining each iMGLs cluster. The number of marker genes per cluster is indicated on the left. **(D)** Sankey diagrams illustrating correspondence between clusters identified in this study and previously published microglial clusters from^43^ Left: projection of all cells; middle: WT cells only; right: KO cells only. **(E–G)** Feature plots and violin plots showing per-cell module scores for E) DLAM-associated gene signatures (from^43^ Supplementary Table 2, Cluster 2, logFC > 0), MHC class II/homeostatic signature (from^43^ Supplementary Table 2, Cluster 3, logFC > 0), and BAM signature (from^48^ Supplementary Table 3, C19) across conditions. UMAPs depict spatial localization of module scores, while violins summarize distribution across experimental groups. **(H)** Flow cytometry validation of disease-associated microglial markers. Box plots show the proportion of TREM2-, CD63-, and CD11C-positive cells in WT and KO iMGLs (n=clone/genotype). In B, E-H p-values were calculated using linear mixed-effects models followed by post hoc pairwise comparisons using estimated marginal means (emmeans). Statistical details are reported in Supplementary File 1.

To determine whether MEF2C-KO iMGLs merely upregulate DLAM genes and whether more cells transition toward a DLAM-like cellular state, we performed single-cell RNA-seq (scRNA-seq) profiling. Since myelin fragments, a lipid-rich stimulus, have been shown to induce the DAM transcriptional response^43^, we challenged both WT and KO iMGLs with human myelin for 24 h. This design yielded four experimental groups: untreated WT and KO (WT_NT, KO_NT) and myelin-treated WT and KO (WT_M, KO_M). We used PIPseq methodology^45^ followed by Seurat scRNAseq pipeline that we used previously^46^. Quality control, integration and clustering were performed before down-sampling cells to the smallest treatment condition for comparison, resulting in 369,730 total cells (Figure 3A; Supplementary Data 3). Cluster annotations were defined based on the enrichment of existing myeloid gene expression signatures, biological pathways, and testing cluster resolutions (Supplementary Figure 2A) to avoid over-clustering. We identified several distinct clusters as evidenced by distinct cluster marker genes that distinguished each cluster from the rest of the cells (Figure 3C, Supplementary Data 3). We found that iMG_0 and iMG_3 showed high expression of DLAM markers such as *CD68, APOE, CTSD* (iMG_0) and *SPP1, TREM2*, and *ITGAX* (iMG_3) (Figure 3A,C). Pathway analysis of iMG_0 and iMG_3 cluster marker genes revealed enrichment in lysosome, endocytosis, efferocytosis (iMG_0), and toll-like receptor and NFkB signaling pathways, lipid and atherosclerosis (iMG_3) suggesting that iMG_0 and iMG_3 most likely represent DLAM states. iMG_1 showed high expression of *HLA-DRB1, HLA-DRA, JAZF1* and *CX3CR1*; pathway enrichment analysis revealed enrichment in ribosome, hematopoietic cell lineage and antigen processing and presentation suggesting this cluster might represent a mixture of homeostatic/surveillance and MHC positive cells. Genes highly expressed in iMG_2 such as *MKI67, STMN1, H2AZ1, HMGN2*, were enriched in cell cycle, DNA replication and spliceosome suggesting a proliferative subset of iMGLs (Supplementary Figure 2B). iMG_7 and iMG_8 showed high expression levels of interferon-related genes (*IFIT1, IFIT3, STAT1*) and cytokine genes (*CCL2, CD83, NFkB1*), respectively suggesting an inflammatory subset of iMGLs (Figure 3C). To validate our cluster assignment, we performed hypergeometric overlap and found significant overlap between cluster marker genes and published myeloid gene sets (Supplementary Data 1). In particular we found strong overlap between iMG_0, iMGL_3 and DAM and LAM cluster marker genes. Similarly, strong overlap was present between MHC positive and homeostatic gene sets and iMG_1, iMG_4 and iMG_8. All proliferative gene signatures strongly overlapped with iMG_2 cluster marker genes indicating faithful recapitulation of major microglial clusters *in vitro* (Supplementary Figure 2C, Supplementary Data 3).

To assess whether the AD risk genes that we previously nominated preferentially localize to specific iMGL clusters, we computed per-cell module scores for putative myeloid AD risk genes^7^. These scores were restricted to the iMG_0 and iMG_7 clusters, both of which exhibit DLAM-like transcriptional signatures. Such enrichment was anticipated, as these genes, like DLAM-associated genes, are involved in endolysosomal processing

To validate these findings, we leveraged associations with AD traits from a previously published large bulk RNA-seq dataset from the ROSMAP cohorts^13^. These bulk RNA-seq data contain transcripts from all parenchymal cells, including microglia and perivascular macrophages. To evaluate the enrichment of our iMGL clusters with genes associated with each AD trait, we calculated the hypergeometric overlap of cluster-specific marker genes with gene sets that were significantly positively (adjusted p < 0.05; log2FC > 0) or negatively (adjusted p < 0.05; log2FC < 0) associated with each of these AD traits. Cluster iMG_5 was enriched for genes that are positively correlated with amyloid-beta pathology, tau pathology, and clinical diagnosis of AD. Consistently, it was also enriched for genes negatively correlated with the slope of cognitive decline where down-regulated genes indicate worsening of cognitive dysfunction (Supplementary Figure 3E). A similar pattern of association with AD traits was detected for iMG_0 and iMG3, two of the DLAM clusters and iMG_7, however, these overlaps were less significant. The color of each box relates to the strength and directionality of each association. Red corresponds with the hypergeometric overlap testing the intersection of cluster marker genes and genes up-regulated (positively associated) with the trait, while blue corresponds with hypergeometric overlap testing intersection between cluster marker genes and genes down-regulated (negatively associated) with the trait.

To determine whether loss of MEF2C and/or myelin exposure had any significant effects on proportion of iMGLs clusters, we used the *propeller* method with adjustment for four independent iMGL clones. Unexpectedly, myelin stimulation did not substantially alter cluster proportions in WT iMGLs, contrasting with prior reports using murine myelin^43^. However, MEF2C loss caused a marked, statistically significant increase (~12%) in the proportion of iMG_0 cells under both basal and myelin-challenged conditions (iMG_0 KO_NT vs WT_NT: 0.1211 [0.0179, 0.2243] p=0.0246; KO_M vs WT_M: 0.1554 [0.0522, 0.2586] p=0.007) (Figure 3B, Supplementary Data 6), accompanied by reductions in homeostatic and proliferative clusters—consistent with bulk RNA-seq findings of downregulated translation and cell-cycle pathways. Notably, myelin exposure did not further increase DLAM cluster in KO iMGLs, as evidenced by similar cluster proportions in KO_NT and KO_M groups (iMG_0 KO_M vs KO_NT: 0.0114 [−.0234, 0.0463] p=0.6831) (Figure 3B). To confirm these changes we performed bulk RNAseq of WT and KO at baseline (NT) and under myelin challenge (M). RRHO analysis detected highly similar changes between WT_M vs WT_NT and KO_M vs KO_NT transcriptomes (Supplementary Figure 1F). Consistently, differential expression investigating interaction between genotype (KO vs WT) and treatment (M vs NT) did not detect any statistically significant genes (Supplementary Figure 1G, Supplementary Data 2) corroborating our scRNAseq. However, GSEA analysis of the entire ranked transcriptome revealed that KO cells react differentially to myelin challenge modifying processes such as cholesterol biosynthesis (negative enrichment) and DNA methylation, HDAC deacetylase histones (positive enrichment) (Supplementary Figure 1H, Supplementary Data 2).

We next mapped our scRNA-seq clusters onto previously published iMGL datasets using *scmap*^47^. DLAM clusters (iMG_0, iMG_3) projected to DAM Clusters 2 and 8, induced by lipid-rich substrates^43^, while iMG_1 projected to antigen-presenting and partially homeostatic clusters found by Dolan *et al.*,^43^ (Figure 3D). Projection of WT and KO subsets separately revealed that most KO iMG_0 cells aligned with DAM Cluster 2, typically induced by synaptosomes, apoptotic neurons, or myelin fragments^43^ (Figure 3D, Supplementary Data 6). Next, we calculated module scores for DAM Cluster 2 and MHCII cluster 3 signatures^43^. Module scoring confirmed increased DAM module expression and reduced MHCII in KO cells regardless of treatment (DAM score KO_NT vs WT_NT: 0.1227 [−0.0173, 0.2627] p=0.0839; Antigen-presentation score KO_NT vs WT_NT: −0.1345 [−0.2297, −0.0393] p=0.0076 (Figure 3E,F). To validate the increased proportion of cells in a DLAM state, we utilized flow cytometry to quantify the percentage of iMGLs expressing canonical DLAM markers such as TREM2, CD63, CD11c (encoded by *ITGAX*). We found an increased percentage of iMGLs expressing TREM2 (32.3 [24.5, 40.1] p < 0.001), CD63 (15 [8.35, 21.7] p < 0.001) and CD11C (10.3 [4.33, 16.3] p=0.006) in KO iMGLs (Figure 3H) consistent with an expansion of the DLAM population. Together, these findings indicate that MEF2C loss induces lysosomal and lipid clearance genes, suppresses homeostatic and proliferative genes, and drives a significant increase of DLAM cluster.

### Loss of MEF2C in iPSC-derived microglia leads to increased expression of border-associated macrophage markers and TREM2 at both transcriptional and surface protein level

Recent studies have shown that MEF2C expression in microglia is required for high *CX3CR1* levels^28^; correspondingly, Mef2c-deficient mouse microglia display reduced *Cx3cr1* expression^29^. However other microglial markers seem to be unchanged in MEF2C-KO iMGLs or mouse microglia^29,31^. To test whether MEF2C loss alters microglial identity, we analyzed expression of canonical microglial markers (*CX3CR1, P2RY12, ITGAM, SALL1*) and border-associated macrophage (BAM) markers (*CD14, FCGR3A, MRC1, CCR2, LYVE1, CD163, CD38*) as these brain macrophages share the same yolk-sac origin. Bulk RNA-seq revealed decreased expression of microglial markers and elevated BAM-associated genes (e.g. *MRC1* (encoding CD206), *CD163*, *SIGLEC1* (encoding CD169), *LYVE1*) in two MEF2C-KO iMGLs lines (Figure 7I). In addition, GSEA analysis revealed significant, positive enrichment of human BAM markers derived from the human brain^48^ and iPSC model^49^ (Figure 7J). Consequently, BAM signature scores were increased in both untreated and myelin-stimulated KO iMGLs as compared to WT iMGLs (KO_NT vs WT_NT: 0.1600 [0.0229, 0.2971] p=0.0256) (Figure 3G). Unlike the human or mouse brain, we did not identify a distinct BAM population in our scRNAseq analysis. Instead, BAM markers were co-expressed within a subset of the DLAM cluster (iMG_0) (Figure 3G). This observation is consistent with the *in vitro* DAM model in which iMGL exposed to phagocytic substrates upregulated canonical BAM markers within the DAM cluster, as reflected by an increased BAM module score in Cluster 2 from Dolan et al.,^43^ (Supplementary Figure 2F).

**Figure 4.**
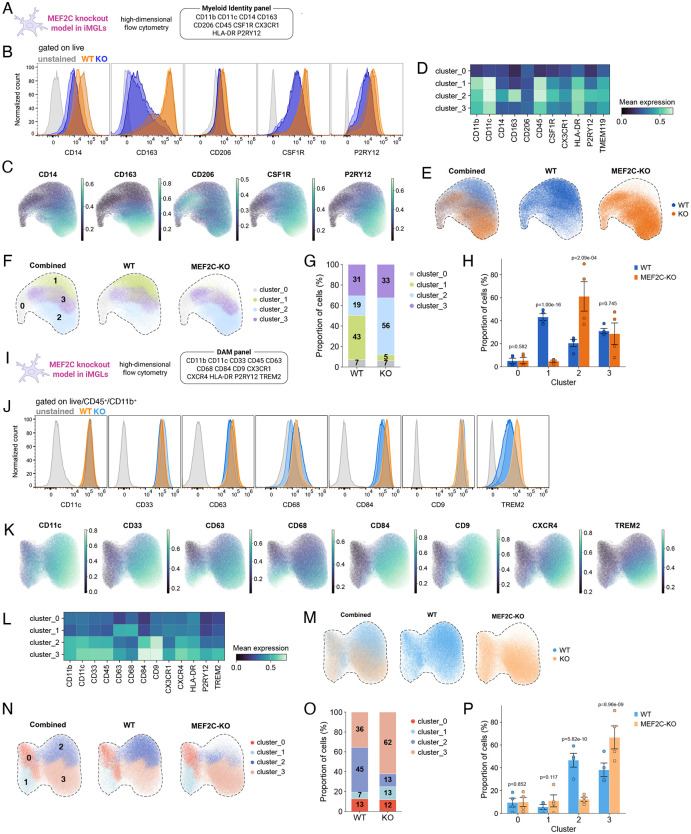
Loss of MEF2C drives expansion of human iPSC-derived microglia toward BAM- and DLAM-like populations revealed by high-dimensional flow cytometry **(A)** Schematic overview of the high-dimensional flow cytometry strategy used to characterize myeloid identity in WT and MEF2C-KO iPSC-derived microglia (iMGLs). **(B)** Representative flow cytometry histograms showing expression of selected macrophage, microglial and BAM-associated markers in WT and MEF2C-KO iMGLs. Grey histograms indicate unstained controls. **(C)** UMAP representation of single-cell cytometry data generated using CytoVI, colored by normalized expression of indicated markers. **(D)** Heat map showing mean marker expression across clusters identified by unsupervised clustering of all live cells. **(E)** CytoVI UMAP embedding of WT and MEF2C-KO cells showing genotype-specific distributions within the combined embedding. **(F)** Cluster identity visualized on the UMAP embedding for combined, WT, and MEF2C-KO datasets. **(G)** Proportion of cells belonging to each cluster in WT and MEF2C-KO iMGLs. **(H)** Quantification of cluster proportions between genotypes. Points represent independent clone replicates. P values were calculated using linear mixed-effects models followed by post hoc pairwise comparisons using estimated marginal means (emmeans); exact P values are shown. **(I)** Schematic overview of a second high-dimensional cytometry panel designed to interrogate disease-associated microglia (DLAM) markers **(J)** Representative histograms showing expression of DLAM-associated markers in WT and MEF2C-KO iMGLs. **(K)** CytoVI UMAP embedding colored by normalized expression of selected DLAM-associated surface markers. **(L)** Heat map showing mean marker expression across clusters identified using the DLAM antibody panel. **(M)** CytoVI UMAP embeddings of combined, WT, and MEF2C-KO datasets. **(N)** Cluster identity projected onto the UMAP embedding for combined, WT, and MEF2C-KO cells. **(O)** Proportion of cells in each cluster across genotypes. **(P)** Quantification of cluster proportions between WT and MEF2C-KO iMGLs. Points represent independent clone replicates. P values were calculated using linear mixed-effects models followed by post hoc pairwise comparisons using estimated marginal means (emmeans); exact P values are shown.

This unexpected increase in BAM markers, coupled with the downregulation of homeostatic and microglia-specific markers, at the transcript level, prompted us to further characterize myeloid populations in KO iMGLs. To this end, we performed high-dimensional flow cytometry analyses to assess protein expression of macrophage, microglial, monocyte, and BAM markers (Figure 4A, Supplementary Figure 3A). We confirmed increased protein expression (manifested by increased geometric mean fluorescence intensity (gMFI)) of BAM-associated markers including CD163 (160,493 [−4,425, 385,412] p=0.136), CD206 (1175 [−89, 3139] p=0.548), and CD14 (16,743 [−604, 35090] p=0.079), in the KO group however none of these markers reach statistical significance mainly due to high variability between clones (Figure 4B, Supplementary Figure 3B,C). In contrast, we did not observe a corresponding decrease in gMFI of canonical microglial markers such as P2RY12 or TMEM119. Instead, several macrophage and microglial markers—including CD45, P2RY12, CSF1R, CX3CR1 and TMEM119—were either unchanged or modestly upregulated at the protein level (CD11B: −7,742 [−24,190, 8,705] p=0.309; CD45: 7,996 [−4,008, 20,000] p=0.326; P2RY12: 5,601 [914, 10,289] p=0.063; CSF1R: 11,725 534, 22,915] p=0.075; TMEM119: 802 [−62, 2,165] p=0.594; CX3CR1: 2,889 [−,232, 8,010] p=0.259) (Figure 4B, Supplementary Figure 3B,C).

We applied CytoVI^50^, a probabilistic deep learning framework for single-cell cytometry analysis, to all live cells to visualize myeloid cell populations and assess differences between WT and KO samples. Following unsupervised clustering, most cells formed a single dominant population characterized by high expression of CD11b and CD45 (clusters_1–3), whereas a minor CD11b^low^CD45^low^ population comprising approximately 7% of total cells was observed in cluster_0 (Figure 4C,F, Supplementary Figure 3D–G). The proportion of CD11b^low^CD45^low^ cells was comparable between WT and KO conditions (Figure 4G,H, Supplementary Figure 3F). Expression of CD45, CD11b, CD11c, and TMEM119 was largely uniform across clusters_1–3 (Figure 4D, Supplementary Figure 3D). In contrast, markers including P2RY12, CD14, CSF1R, and CD163 displayed graded expression patterns, with the highest levels observed in cluster_2 (Figure 4C,F). Cluster_3 exhibited the highest expression of HLA-DR whereas cluster_1 was characterized by high expression of CD11c, CD11b, and CD45 but low expression of CSF1R, TMEM119, CX3CR1, and HLA-DR, and lacked detectable CD14 and CD163 expression (Figure 4C,D,F). Analysis of genotype-specific cluster distributions revealed that more than half of the KO population (56%) localized to cluster_2, defined by high expression of CD14 and CD163, whereas only 19% of WT cells mapped to this cluster (Figure 4G,H, Supplementary Figure 3F). Conversely, 43% of WT cells were found in cluster_1, which displayed low expression of BAM-associated markers, compared to only 5% of KO cells. Unexpectedly, cluster_2 also showed high expression of P2RY12, a canonical marker of homeostatic microglia. We confirmed that CD14/CD163 (BAM markers) and P2RY12/CSF1R are co-expressed in these cells (Supplementary Figure 3E), suggesting the presence of a hybrid iMGL state with features of both microglial and BAM-like identities.

To validate the scRNA-seq findings indicating an increased proportion of DLAM clusters in KO iMGLs, we applied an orthogonal approach using high-dimensional flow cytometry with a second antibody panel targeting DLAM-associated markers (Figure 4I). Live CD45^+^CD11b^+^ cells were first gated (Supplementary Figure 3A). Consistent with the transcriptomic analysis, several DLAM markers exhibited increased expression in KO iMGLs, reflected by higher geometric mean fluorescence intensity (gMFI), although only TREM2 reached statistical significance (CD63 (13,787 [−0,474, 38,047] p=0.211), CD68 (4,111 [−,070, 11,291] p=0.209), CD9 (4,593 [−93,555, 202,741] p=0.956), CD84 (62,148 [−7,669, 161,965] p=0.176), CXCR4 (2,402 [−,610, 7,415] p=0.279, and TREM2(5,725 [124, 11,326] p=0.046)) (Figure 4J, Supplementary Figure 3I). Unsupervised clustering identified four populations, with CD11b, CD11c, and CD45 uniformly expressed across all clusters. Cluster_0 showed low or absent expression of DAM markers, whereas cluster_3 was characterized by high expression of canonical DLAM-associated surface proteins, including TREM2, CD9, CXCR4, and CD84. Notably, two additional DLAM-associated markers, CD63 and CD68, were highly expressed in cluster_1, suggesting the presence of two distinct DLAM-like populations, consistent with the heterogeneity observed in our scRNA-seq analysis (Figure 4K,L). Cluster_2 was defined by high expression of CD9, CD11c, and HLA-DR, although their peak expression was observed in cluster_3 (Figure 4K,L,N). Comparison of cluster distributions between genotypes revealed marked differences in clusters_2 and 3. In WT iMGLs, cluster_2 represented the dominant population (42%), whereas this cluster was substantially reduced in KO iMGLs (13%). Conversely, KO iMGLs exhibited a pronounced shift toward cluster_3, resulting in an expanded TREM2^high^ population (62%). Cluster_1 (CD63^high^CD68^high^) was also modestly enriched in KO cells (13%) compared to WT cells (7%) (Figure 4O,P, Supplementary Figure J).

Altogether, these orthogonal surface proteomic analyses corroborate the transcriptomic findings and demonstrate that loss of MEF2C drives a shift in iMGL toward BAM- and DLAM-like subpopulations, characterized by increased expression of BAM markers and expansion of a TREM2^high^ population.

### MEF2C controls lysosomal and cholesterol clearance processes in iMGLs

**Figure 5.**
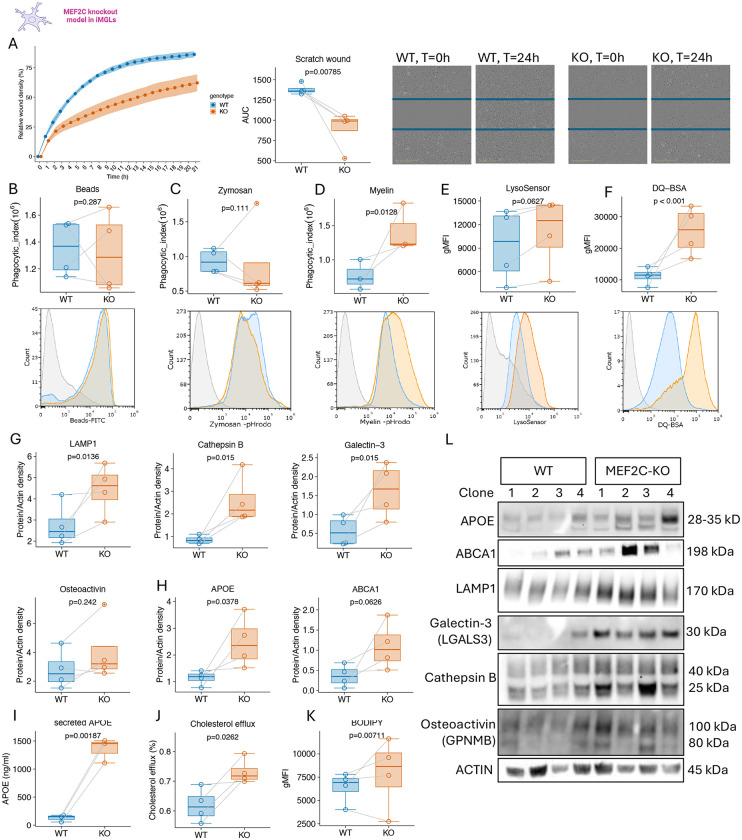
MEF2C loss enhances phagocytic, lysosomal, and cholesterol efflux functions in human iPSC-derived microglia (iMGLs) **(A)** Scratch-wound migration assay in WT and KO iMGLs. Left, quantification of relative wound density over time; middle, area-under-the-curve (AUC) comparison between genotypes; right, representative images exported from Incucyte S3. **(B-D)** Phagocytic capacity assessed by uptake of fluorescent beads, pHrodo labelled-zymosan, and pHrodo labelled-myelin particles. Box plots show the mean of phagocytic index (Methods) for each substrate in WT and KO iMGLs, with representative flow cytometry histograms shown below. **(E-F)** Lysosomal activity measured using LysoSensor and DQ–BSA assays. Box plots show geometric mean fluorescence intensity (gMFI), with representative flow cytometry histograms shown below. **(G-H)** Immunoblot analysis of lysosomal and cholesterol-associated proteins in WT and KO iMGL. Box plots show protein density over actin density. Representative blots shown in L). **(I)** Quantification of secreted APOE in culture supernatant by APOE ELISA. Box plot shows the mean of APOE concentration. **(J)** Quantification of cholesterol efflux to HDL cholesterol acceptor. Box plot show the mean of cholesterol efflux **(K)** Quantification of BODIPY^+^ neutral lipid droplets in WT and KO iMGLs. Box plots show the geometric mean fluorescence intensity (gMFI) of BODIPY signal measured in flow cytometry **(L)** Representative immunoblots corresponding to G,H). Uncropped western blot images are shown in Supplementary Figure 8. For all box plots, points represent independent clone replicates (n=4/genotype), with paired measurements connected by lines. P values were calculated using linear mixed-effects models followed by post hoc pairwise comparisons using estimated marginal means (emmeans); exact P values are shown.

To validate the functional impact of MEF2C loss on the biological processes identified through the pathways and clustering analyses described above, we performed a series of functional assays. For all quantitative analyses, we report the estimated mean difference and specify the variable tested in the corresponding linear mixed-effects models. Migration capacity, assessed using a scratch-wound assay, was markedly reduced in MEF2C-KO iMGLs (area under curve, AUC: −492 [−799, −184] p=0.008) (Figure 5A). This phenotype is consistent with the downregulation of actin cytoskeleton and adhesion genes identified in the transcriptomic dataset (Figure 2A), suggesting impaired cytoskeletal dynamics underlie the reduced migratory potential. To examine endolysosomal processing, we employed phagocytic substrates including beads, zymosan, and myelin fragments. While MEF2C-KO iMGLs exhibited a non statistically significant decrease in phagocytosis of latex beads and zymosan particles (Phagocytic index: Beads: −101,074 [−294,842, −92,695] p=0.287; Zymosan: −174,522 [−392,420, 43,375] p=0.111), they exhibited a statistically significant increase in phagocytosis of myelin fragments (487,803 [120,536, 855,069] p=0.0128), compared to WT iMGLs (Figure 5B–D). We next evaluated lysosomal function to examine degradation capacity and validate increased RNA level of lysosomal genes (e.g. cathepsins) (Figure 2B,D). MEF2C-KO iMGLs displayed increased lysosomal acidification, indicated by higher gMFI values of the LysoSensor dye, which fluoresces proportionally to acidity (lower pH) (2,079 [−51, 4,309] p=0.063) (Figure 5E). Consistently, lysosomal proteolytic activity was elevated, as demonstrated by enhanced fluorescence of digested BSA in the DQ-BSA assay (13,579 [6,868, 20,290] p=0.001) (Figure 5F). These findings are in agreement with transcriptomic enrichment of lysosomal genes, including cathepsins (*CTSB, CTSZ, CTSL*) and ATPases (*ATP2C1*, *ATP6V1B2*). Moreover, protein-level analyses confirmed upregulation of lysosomal markers such as LAMP1 (protein density: 4,709 [1,588, 7,830] p=0.0136), Cathepsin B (CTSB) (4,191 [1,297, 7,084] p=0.015), Galectin-3 (LGALS3) (2,495 [632, 4,357] p=0.020), and Osteoactivin (GPNMB) (3,179 [−,231, 9,589] p=0.242) in MEF2C-KO iMGLs, further supporting enhanced lysosomal activity and increased DLAM abundance (Figure 5G,L).

To determine whether MEF2C loss also alters cholesterol metabolism, we examined NR1H2/3-mediated cholesterol transport pathways. MEF2C-KO iMGLs displayed increased expression of key cholesterol efflux mediators ABCA1 (protein density: 2.542 [−83, 5,268] p=0.062) and APOE (Figure 5H,L), with elevated APOE levels detected in both total cell lysates (protein density: 4,025 [328, 7,722] p=0.0378) and culture supernatants (ng/ml: 1,263 [854, 1,673] p=0.002) (Figure 5I). To assess intracellular lipid handling, we quantified neutral lipid droplet accumulation and cholesterol efflux. Cholesterol efflux assays demonstrated an increased fraction of cholesterol exported to HDL transporters (fluorescence: 0.738 [0.127, 1.35] p=0.026) (Figure 5J). BODIPY staining revealed increased accumulation of neutral lipid droplets in MEF2C-KO iMGLs (gMFI: 2,427 [763, 4,091] p=0.0071) (Figure 5K). Altogether these data suggest that MEF2C restrains lysosomal and cholesterol clearance processes in iMGLs.

### Increased chromatin accessibility for master regulators of lysosomal and cholesterol clearance in MEF2C-KO iMGLs

**Figure 6.**
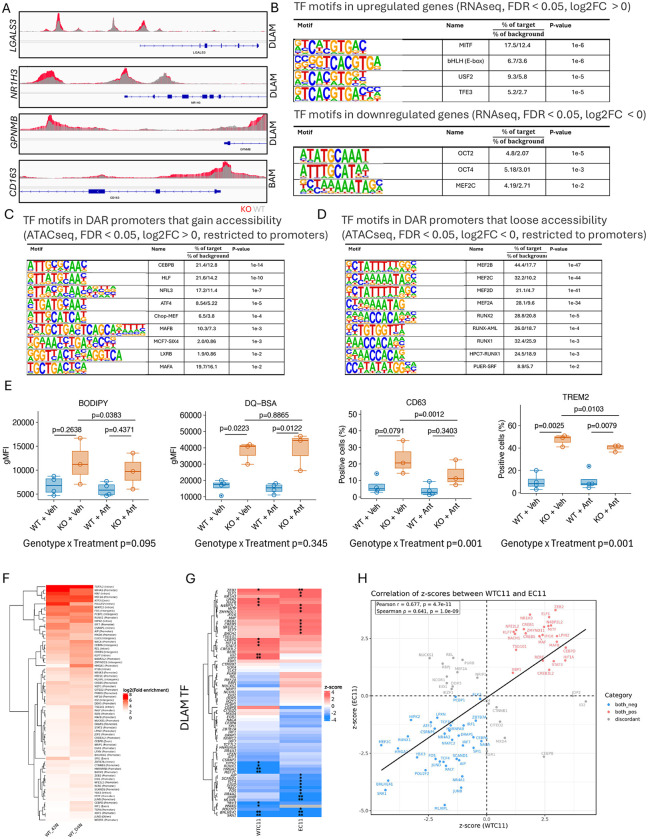
MEF2C coordinates a transcriptional and chromatin regulatory program underlying disease-associated microglial states **(A)** Genome browser tracks showing ATAC-seq signal at representative DLAM and BAM genes in WT and KO iMGLs in WTC11 line **(B)** Transcription factor (TF) motif enrichment analysis in genes significantly upregulated (top) or downregulated (bottom) in KO iMGLs (Supplementary Data 2 RNA-seq, adj.p.val < 0.05). Tables report enriched TF motifs, associated TFs, target-to-background ratios, and enrichment p-values. **(C)** TF motif enrichment in promoter-associated differentially accessible regions (DARs) that gain chromatin accessibility upon MEF2C loss (ATAC-seq, FDR < 0.05, log2FC > 0), restricted to promoter regions. **(D)** TF motif enrichment in promoter-associated DARs that lose chromatin accessibility upon MEF2C loss (ATAC-seq, FDR < 0.05, log2FC < 0), highlighting depletion of MEF2C and related regulatory motifs. **(E)** Functional perturbation of MEF2C-regulated pathways using pharmacological LXRα/β antagonist (GSK2033). Box plots show neutral lipid droplets accumulation (BODIPY), lysosomal proteolytic activity (DQ–BSA), and surface expression of DLAM markers (CD63 and TREM2) across WT and KO iMGLs treated with vehicle or LXRα/β antagonist. Linear mixed-effects models were used to assess genotype, treatment, and interaction effects followed by post hoc pairwise comparisons using estimated marginal means (emmeans); exact p-values are shown. **(F)** Heatmap showing fold enrichment of DLAM-associated transcription factors—previously nominated in^20^ —among direct MEF2C binding targets identified by MEF2C ChIP–seq^31^(Supplementary Data 1). Values represent log2 fold enrichment of MEF2C-bound promoters or regulatory regions for each DLAM transcription factor across two WT samples. **(G)** Heatmap of log2(Fold change) values comparing KO vs WT from RNAseq for DLAM-associated transcription factors across independent iMGL lines (WTC11 and EC11^31^). Stars represents adj.p.val form RNAseq * p < 0.05, ** p < 0.0, *** p < 001 (Supplementary Table 2) **(H)** Correlation analysis of TF log2(Fold change) between WTC11 and EC11 iMGL lines. Scatter plot shows concordant and discordant TF activity changes, with Pearson and Spearman correlation coefficients indicated.

To further explore the mechanisms underlying DLAM activation, we performed ATAC-seq in MEF2C-KO and WT iMGLs. We identified 9,706 differentially accessible regions (DARs) (Supplementary Data 5). Surprisingly, only a few DLAM genes gained accessibility in the promoter region, including *LGALS3*, *GPNMB*, and *NR1H3* (Figure 6A), whereas transcriptomic analyses revealed broader upregulation across DLAM-associated genes (Figure 2D). We hypothesized that loss of MEF2C may reshape chromatin to facilitate recruitment of other TFs that orchestrate the DLAM response, suggesting that MEF2C acts upstream of other DLAM-related regulators. To identify TFs potentially driving this response, we used HOMER (*findMotifs.pl*)^51^ to analyze enriched motifs in promoters of upregulated genes identified in bulk RNAseq. Among the top hits, MITF—a master regulator of lysosomal clearance and a known DAM transcription factor^20,43^—showed the strongest motif enrichment, suggesting its involvement in the lysosomal phenotype of MEF2C-KO iMGLs (Figure 6B). We next performed stratified motif enrichment analysis to separately examine TF motif enrichment in promoters gaining (positive DARs) or losing (negative DARs) accessibility. CEBPB, TFEB, MITF, and LXRβ motifs were enriched among regions gaining accessibility, indicating that these TFs may drive transcriptional upregulation of lysosomal and lipid metabolism genes (Figure 2D). In contrast, promoters losing accessibility were enriched for motifs recognized by RUNX1 and the MEF2 family, consistent with the loss of MEF2C function (Figure 6D). Together, these results suggest that MEF2C loss remodels chromatin to favor binding of DLAM TFs, including LXR and MITF/TFE family members, thereby explaining the transcriptional and functional reprogramming observed in MEF2C-KO iMGLs. Among these TFs, *NR1H2* and *NR1H3*, encoding LXRβ and LXRα respectively, are of particular interest. Both are master regulators of cholesterol efflux and reside within AD risk loci (*NR1H3* within the *SPI1* locus^10,52^ and *NR1H2* within the *SIGLEC11* locus^53^). Notably, the *NR1H3* promoter gained accessibility in MEF2C-KO iMGLs, consistent with increased transcript levels (Figure 2D). These findings suggest that MEF2C may act as a repressor of LXRα/β, analogous to BHLHE40/41, which we and others previously showed inhibit LXR signaling^20,54^. To test this hypothesis, we treated iMGLs with an LXRα/β antagonist (GSK2033, 1μM, 24h). Pharmacologic blockade of LXRα/β partially reduced BODIPY^+^ lipid droplet accumulation and expression of CD63 and TREM2 as evidenced by significantly lower BODIPY gMFI (−1,874 [−3,619, −130] p=0.0383) and reduced percentage of positive cells expressing DLAM markers (CD63: −9.35 [−13.25, −5.45] p=0.001; TREM2: −6.98 [−11.7, −2.28] p=0.103) in KO + GSK2033 as compared to KO + Veh iMGLs (Figure 6E). As expected, inhibition of LXRα/β did not affect lysosomal proteolysis, which remained elevated in KO cells after treatment with an antagonist as compared to vehicle treated KO (1,600 [−,636, 9,835] p=0.886) (Figure 6E). These results indicate that a subset of the DLAM phenotype is mediated, at least in part, through LXRα/β signaling; however, the modest reduction (20–30%) suggests that other TFs cooperate with LXRα/β to fully drive the DLAM program in MEF2C-deficient iMGLs.

To determine which TFs may be regulated by MEF2C, we checked how many DLAM TFs that we have previously nominated^20^ are bound by MEF2C utilizing MEF2C ChiPseq data (Supplementary Data 1). We found that 67 out of 74 DLAM TF are direct targets of MEF2C, out of which 47 are bound in promoters suggesting direct regulation (Figure 6F). Next, we checked the expression level of these 67 DLAM TFs that are also MEF2C targets and found that nearly one third is upregulated, another one third is downregulated and the rest are unchanged in two MEF2C-KO RNAseq datasets from WTC11 and EC11 lines (Figure 6G). Correlation analysis revealed highly concordant changes in TF expression between WTC11 and EC11 lines (ρ=0.641, p=1.0e-09) including upregulation of *NR1H3*, *MITF* and *MAFB* and downregulation of *BHLHE41*, *POU2F2*, and *RUNX1* (Figure 6H). Taken together these data suggest that MEF2C may be upstream of other DLAM TF and its downregulation may be the first step toward transition to the DLAM state.

### Partial reduction of MEF2C in iMGLs is sufficient to induce DLAM transcriptional responses

To determine whether partial MEF2C loss is sufficient to recapitulate the phenotypes observed in MEF2C-KO iMGLs, we established a model of MEF2C reduction in iMGLs using four different iPSC lines, including two female and two male lines, to account for biological sex (Supplementary Figure 4A). iPSCs were differentiated into iMGLs, and MEF2C was transiently reduced via siRNA during the final two days of maturation, with scrambled RNA (SCR) as a control. Reduction of MEF2C was confirmed at both the transcript and protein level (transcript log2FC: −0.654 [−0.987, −0.321] p<0.001; protein density: (−6,669 [−12,043, −1,295] p=0.021) (Supplementary Figure 4B,C). RT-qPCR analysis revealed increased expression of core DLAM genes, including *APOE* (log2FC: 0.508 [0.175, 0.841] p=0.006), *LGALS3* (log2FC: 0.509 [0.32, 0.698] p<0.001), *GPNMB* (log2FC: 0.226 [0.0538, 0.397] p=0.014), and *ABCA1* (log2FC: 0.441 [0.015, 0.867] p=0.0435) among other markers, across all four KD iMGL lines compared to SCR controls (Supplementary Figure 4B). Protein-level validation confirmed statistically significant elevated expression of ABCA1 (protein density: 3,128 [551, 5,704] p=0.024) and Galectin-3 (*LGALS3*) (protein density: 2,814 [751, 4,877] p=0.014), and non statistically significant increase of Osteoactivin (*GPNMB*) (protein density: 1,841 [−,287, 5,970] p=0.306), LAMP1 (protein density: 1,080 [−,153, 4,313] p=0.470), and APOE (protein density: 531 [−,812, 4,875] p=0.7892] (Supplementary Figure 4C). In summary, partial loss of MEF2C induces expression of DLAM-associated markers recapitulating changes observed in KO iMGLs.

### Partial reduction of MEF2C in THP-1 macrophages is sufficient to induce transition to a transcriptional and functional DLAM state

**Figure 7.**
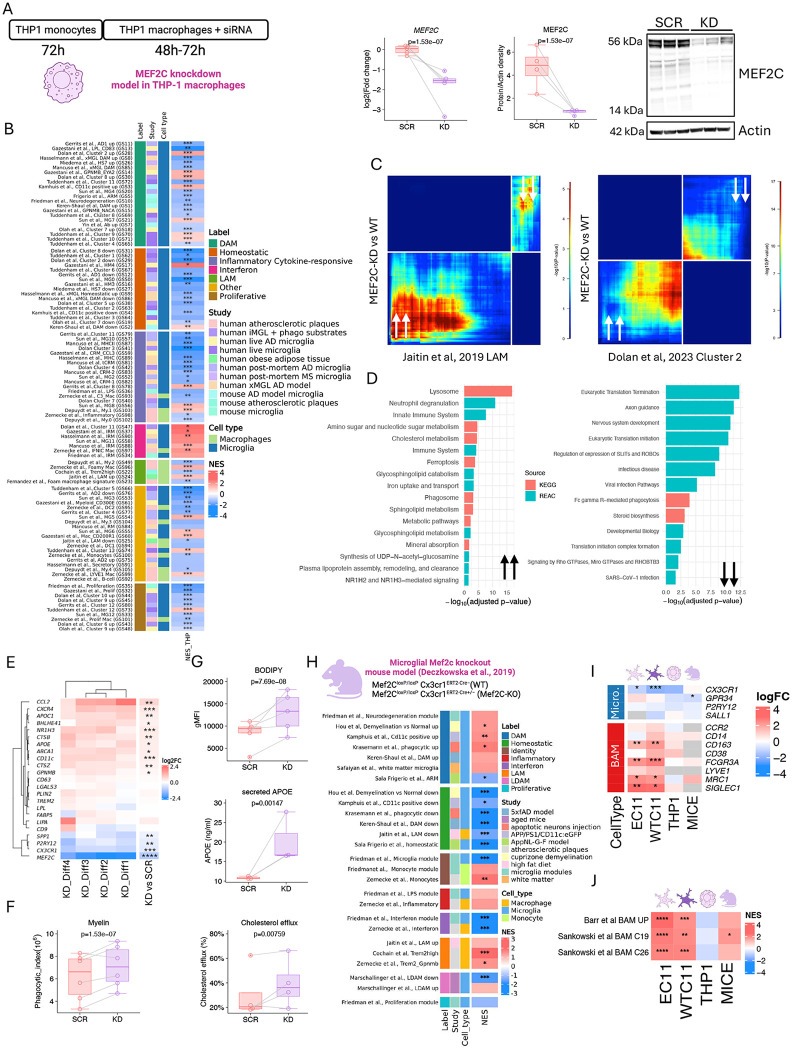
MEF2C knockdown in THP-1 macrophages and Mef2c knockout in mouse microglia recapitulate increased DLAM transcriptional and functional responses **(A)** Experimental schematic of MEF2C knockdown in THP-1–derived macrophages using siRNA. MEF2C expression was assessed by qPCR and protein quantification. **(B)** Enrichment of published myeloid gene sets following MEF2C knockdown in THP-1 macrophages in KD THP-1 macrophages. Heatmap shows normalized enrichment scores (NES) across myeloid signatures derived from human and mouse studies annotated by study source and cell type (Supplementary Data 4). **(C)** Rank–rank hypergeometric overlap (RRHO) analysis comparing the MEF2C knockdown transcriptome with the DAM cluster from^43^, Supplementary Table 2, Cluster 2) and LAM from^17^ (Dataset S6, FDR < 0.05). Heatmaps depict regions of significant concordant upregulation and downregulation between datasets. **(D)** Pathway enrichment analysis of genes contributing to the most significant RRHO overlap regions. Bar plots show enriched KEGG and Reactome pathways for concordantly upregulated (left) and downregulated (right) gene sets. **(E)** qPCR analysis of core DLAM genes in THP-1 macrophages following MEF2C knockdown. The heatmap shows log2(fold change) relative to scrambled control (SCR) within each differentiation replicate and the overall KD versus SCR effect size. Asterisks denote statistical significance. **(F-G)** Functional validation of MEF2C knockdown effects in THP-1 macrophages. Box plots show phagocytosis of myelin, secretion of APOE, neutral lipid accumulation (BODIPY), and cholesterol efflux capacity in SCR and MEF2C-KD cells. **(H)** Cross-species comparison of MEF2C-regulated myeloid programs. Heatmap summarizes enrichment of myeloid gene signatures in a microglia-specific Mef2c knockout mouse model^29^. Reprocessed bulk RNAseq data can be found in Supplementary Data 4. **(I)** Heatmap showing log2(Fold change) from RNAseq of selected microglia and BAM genes across human iMGLs, THP-1 macrophages, and mouse microglia following MEF2C perturbation. Stars represents adj.p.val form RNAseq * < 0.05, ** < 0.01 **(J)** Enrichment of published BAM gene sets across human iMGLs, THP-1 macrophages, and mouse microglia following MEF2C perturbation. Stars represents FDR-corrected p value form GSEA In A and E,F,G points represent individual THP-1 macrophage differentiation; paired measurements are connected. p-values were calculated using linear mixed-effects models followed by post hoc pairwise comparisons using estimated marginal means (emmeans); exact p-values are shown.

To extend our findings to a model resembling peripheral macrophages, we utilized THP-1 cells differentiated into macrophages. MEF2C expression was transiently reduced by siRNA-mediated knockdown (KD), with scrambled RNA (SCR) serving as control. We achieved approximately 60% reduction of MEF2C at both transcript and protein levels in KD cells compared to SCR controls (transcript log2FC: −1.9 [−2.29, −1.51] p<0.001; protein density: −15,901 [−20,081, −11,721] p<0.001) (Figure 7A). Bulk RNA-seq revealed a limited number of significantly up- or downregulated genes (Supplementary Data 3). However, RRHO analysis revealed strong concordance between THP-1 macrophages with reduced level of MEF2C and human iMGLs with genetic inactivation of MEF2C (Supplementary Figure 6A). GSEA identified positive enrichment of seven DLAM gene sets, particularly those generated from live microglia from either iMGLs or human biopsies^13,43^. Fourteen DLAM gene sets, particularly those from mouse models^15,35,55^, were negatively enriched. We observed negative enrichment for homeostatic and proliferative gene sets, and strong positive enrichment of interferon response pathways (Figure 7B). Correlation analyses between KD and previously defined DLAM clusters—Cluster 2 (DAM) from^43^ and LAM from^17^ — showed concordant transcriptional changes, particularly in genes associated with lysosomal activity, efferocytosis, and endocytosis (Figure 7C,D). To verify if the core DLAM genes are induced in THP-1 macrophages with reduced level of MEF2C, we performed qPCR analysis. We found increased expression of *APOE* (log2FC: 0.588 [0.137, 1.04] p=0.018), *ABCA1* (0.603 [0.17, 1.03] p=0.013)*, CTSB* (0.644 [0.22, 1.07] p=0.009)*, NR1H3* (0.707 [0.41, 1.00] p=0.0008)*, GPNMB* (0.305 [0.098, 0.51] p=0.010) and reduced expression of *CX3CR1* (−0.837 [−1.15, −0.52] p=0.0004) and P2RY12 (−0.703 [−1.17, −0.23] p=0.009) corroborating our partial knockdown in iMGLs (Figure 7E). To functionally validate these transcriptomic findings, we performed the same assays as in KO iMGLs. MEF2C-KD THP-1 macrophages showed increased phagocytosis of myelin fragments (Phagocytic index: 347,135 [576,346, 1,117,925] p<0.001) (Figure 7F), elevated APOE secretion (ng/ml: 8.18 [3.59, 12.8] p=0.002), higher BODIPY^+^ lipid droplet accumulation (gMFI: 4,834 [3,434, 6,234] p<0.001), and enhanced cholesterol efflux (fluorescence: 1.5 [0.435, 2.58] p=0.008) (Figure 7G). These results indicate that partial MEF2C reduction is sufficient to drive DLAM-like transcriptional and functional reprogramming, supporting a dose-dependent regulatory role of MEF2C in macrophage DLAM state induction.

### Mouse microglia lacking MEF2C showed increased DLAM responses

As we have previously shown, MEF2C was also nominated as a regulator of DLAM responses in mouse networks^20^. To test whether this role of MEF2C is conserved across species, we leveraged transcriptomic data from mice lacking MEF2C specifically in microglia^29^. We first reanalyzed the bulk RNA-seq data comparing KO versus WT microglia (Supplementary Data 4). RRHO analysis comparing mouse and human (WTC11 iMGL) transcriptomes revealed strong concordance among the most significantly up- and downregulated genes, along with a moderate but highly significant positive correlation at the pathway level (ρ = 0.22, p = 6.08 × 10^−18^). (Supplementary Figure 6B). Next, we performed GSEA using mouse myeloid gene sets extracted from mouse models of amyloidosis^15,34,35,55^, aging^56^, demyelination^57^, atherosclerosis^18,40^, and high fat diet^17^ (Supplementary Data 4). These analyses revealed a positive enrichment of several DLAM-associated mouse gene signatures and a negative enrichment of homeostatic gene sets (Figure 7H). Notably, we also observed a negative enrichment of mouse microglial module genes and a positive enrichment of BAM gene sets in MEF2C-deficient microglia, suggesting a conserved role for MEF2C in establishing microglial transcriptional programs similar to that observed in human iMGLs (Figure 7I,J).

### Loss or reduction of MEF2C does not lead to a significant exacerbation of inflammatory response to LPS

Two recent studies using MEF2C-deficient human microglia reported increased proinflammatory cytokine levels, suggesting that MEF2C restrains microglial immune activation as evidenced by increased proinflammatory cytokine secretion^30,31^. While minimal differences were observed in induction of inflammatory response at baseline between WT and KO microglia, MEF2C loss reportedly led to exaggerated inflammatory responses following LPS or TNF stimulation. To evaluate this in our human model, we treated MEF2C-KO and WT iMGLs with LPS (50ng/mL, 24h) and performed bulk RNA-seq. Both genotypes responded to LPS stimulation, showing upregulation of inflammatory cytokines such as *CCL2* and *CD80* and LPS-induced transcription factors such as *STAT1* and *SOCS3* (Supplementary Figure 4A). However, the magnitude of LPS-induced changes was smaller in KO iMGLs as compared to WT, the pattern of transcriptional changes was highly similar between genotypes, as evidenced by strong concordance between the WT_LPS vs WT_NT and KO_LPS vs KO_NT contrasts in rank rank hypergeometric overlap test (Supplementary Figure 5B). Importantly, no differentially expressed genes were detected in the genotype × treatment interaction analysis (Supplementary Figure 5C), indicating that MEF2C loss does not exacerbate the inflammatory response to LPS in iMGLs. Lastly, we confirmed these findings at the protein level by measuring IL-1β, TNFα, IL-6, and CCL2 concentrations at baseline and following LPS stimulation. We detected no significant genotype × treatment interaction for any of the cytokines tested. However, we observed a trend toward increased IL-6 levels in the KO_LPS group compared to WT_LPS (pg/ml: 11.09 [−7.57, 29.8], p = 0.349). Consistent with our transcriptomic observations, CCL2 levels were markedly elevated in MEF2C-deficient cells both at baseline (pg/ml: KO vs WT in NT: 341.4 [−500.6, 1183], p = 0.581) and following LPS stimulation (pg/ml: KO–WT in LPS: 204.9 [−637, 1047], p = 0.859). These effects did not reach statistical significance, largely due to substantial inter-clone variability (Supplementary Figure 5D).

To confirm these findings in an independent system, we used THP-1 macrophages transiently depleted of MEF2C and stimulated them with LPS (50ng/mL, 24h) or IFNγ (50ng/mL, 24h). As in iMGLs, we observed comparable induction of proinflammatory cytokines between MEF2C-KD and control (SCR) cells treated with LPS (Supplementary Figure 5E). The only exception was *CCL2*, which showed a significant genotype × treatment interaction in LPS challenge (β=0.98 [0.64, 1.33] p<0.001): *CCL2* expression was elevated in KD cells at baseline and did not further increase upon LPS stimulation compared to SCR_LPS-treated cells (log2FC: KD_NT vs SCR_NT: 1.10 [0.76, 4.46] p<0.001; KD_LPS vs SCR_LPS: 0.126 [−0.238, 0.49] p=0.901)(Supplementary Figure 5E). Notably, IFNγ enhanced the expression of *CCL2*, *IL6*, and *TNFA* in MEF2C-KD THP-1 macrophages compared to SCR cells treated with IFNγ, although the increase reached statistical significance only for *CCL2* (log2FC KD_IFNγ vs SCR_IFNγ: 0.714 [0.177, 1.25] p=0.004). Interestingly, CCL2 has been implicated as a marker of the proinflammatory DAM subset and functions as a chemoattractant for monocytes^37,58^. This observation suggests that MEF2C deficiency may selectively enhance CCL2-driven signaling pathways, potentially influencing blood–brain barrier permeability and monocyte recruitment.

### MEF2C-regulated enhancers link epigenetic activity to AD risk

**Figure 8.**
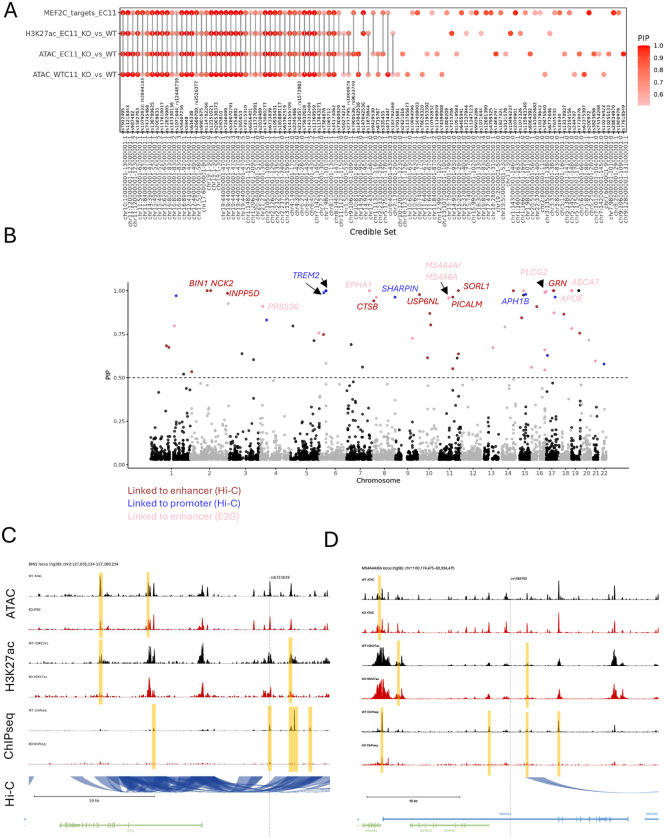
MEF2C-regulated microglial regulatory elements harbor fine-mapped AD risk variants **(A)** Integration of fine-mapped AD GWAS credible sets with MEF2C-dependent microglial regulatory landscapes. Each column represents an AD GWAS credible set^53^, with dots indicating posterior inclusion probability (PIP) for individual variants. Rows denote overlap with MEF2C ChIP–seq targets(raw data:^31^, reprocessed data: Supplementary Data 1,5), H3K27ac-marked regions (raw data:^31^, reprocessed data: Supplementary Data 5), and ATAC-seq peaks ((raw data:^31^ (EC11) and this study (WTC11), reprocessed data: Supplementary Data 5) that are differentially accessible upon MEF2C knockout in human iPSC-derived microglia (EC11 and WTC11 lines). Color intensity reflects PIP score. **(B)** Genome-wide distribution of fine-mapped AD GWAS variants plotted by chromosomal position and PIP. Variants exceeding the high-confidence threshold (PIP ≥ 0.5, dashed line) are highlighted and annotated with linked candidate genes through HiC data^6^. Colors indicate the type of regulatory connection, including promoter or enhancer linkage inferred from Hi-C–based chromatin interactions or enhancer–gene links using e2g portal. **(C-D)** Representative genome browser tracks illustrating MEF2C binding, chromatin accessibility, and enhancer activity at selected AD risk loci, demonstrating colocalization of high-PIP variants with MEF2C-regulated microglial regulatory elements. Yellow spikes indicate statistically significant differentially accessible, acetylated bound regions C) BIN1 locus, D) MS4A4A/6A locus.

To identify candidate AD causal variants residing within MEF2C-regulated cis-regulatory elements, we performed fine-mapping informed by functional epigenetic annotations using PolyFun followed by a sum of single effects (SuSiE)^59^. We used four complementary datasets, including ATACseq from two iMGL lines (WTC11 and EC11^31^), H3K27ac profiles (EC11^31^) and MEF2C ChIPSeq (EC11^31^). In each case we restricted the AD GWAS signal^53^ to differentially accessible regions and differentially acetylated H3K27ac peaks (KO vs WT), and MEF2C binding sites (FDR < 0.001) (see Methods). This approach identified 103 fine-mapped variants with posterior inclusion probability (PIP) > 0.5 across the four datasets (union), of which nearly half (43 variants) were shared by all four datasets (intersection) (Figure 8A, Supplementary Data 5). Notably, these intersected SNPs had PIP > 0.8 demonstrating only the most plausible SNPs were shared.

To link fine-mapped variants to candidate causal genes, we leveraged microglia Hi-C–based enhancer–gene interaction datasets^6,60^. Because individual variants are often mapped to multiple candidate genes, we further refined this set by prioritizing genes previously nominated as likely causal in AD^6,7,53,61^. This analysis highlighted multiple high-confidence variants linked to lysosomal genes (*BIN1, INPP5D, CTSB, PICALM, SORL1, GRN*), lipid-associated genes (*APOE, ABCA7, TREM2*), and MS4A family members (*MS4A4A/MS4A6A*) (Figure 8B). Interestingly the APOE and TREM2 SNPs are cis-regulatory variants in low linkage disequilibrium with the known missense variants at these loci.

Our fine-mapping analysis nominated rs6733839 as a highly credible causal variant (PIP = 1) residing within a microglia-specific enhancer linked to *BIN1*^6^ (Figure 8C). Deletion of this enhancer resulted in downregulation of BIN1 expression only in iMGLs but not in iPSC-derived astrocytes and neurons^6^. Consistent with that observation the expression of *BIN1* is downregulated in KO iMGLs (WTC11 and EC11 lines) (Supplementary Data 2). Another interesting high-confidence variant (PIP = 0.95) that we identified is rs1582763 that has been linked to *MS4A4A/6A* genes (Figure 8D). This variant is in high LD with another variant nominated in this locus rs636317 (r2 = 0.8435, D’ = 0.9909, p<0.0001), that has been shown to disrupt CTCF binding^7^. *MS4A4A/6A* are direct targets of MEF2C, regions linked to *MS4A4A/6A* are gaining accessibility and are more acetylated in KO iMGLs (Figure 8D) consequently leading to upregulation of these genes in two iMGL KO lines (Supplementary Data 2).

Altogether these data present mechanistic evidence that MEF2C-regulated cis-regulatory elements affect the expression of AD risk genes and thus may modulate disease susceptibility.

### Loss of MEF2C in iMGL co-cultured with AD neurons and astrocytes increase the CD9 DLAM population and reduce Aβ42/40 ratio

**Figure 9.**
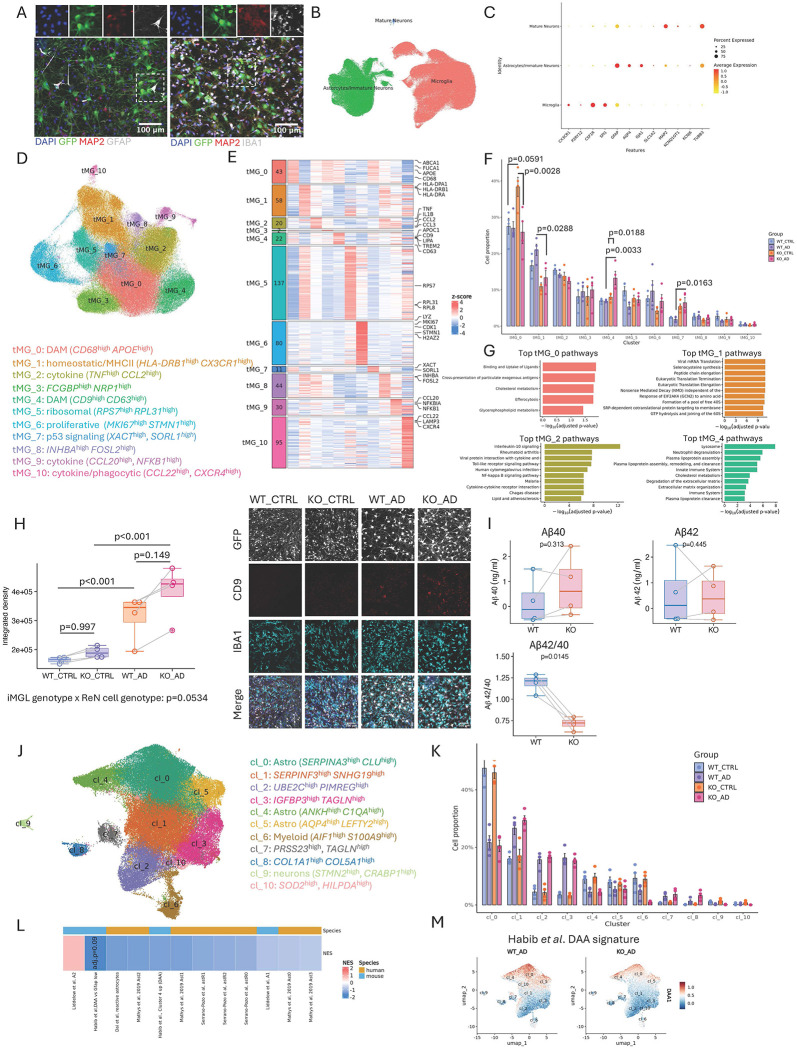
MEF2C loss in iMGLs promotes DLAM states involved in amyloid clearance in an AD neuron–astrocyte co-culture system **(A)** Representative immunofluorescence images of Alzheimer’s disease (AD) ReN-derived neuron–astrocyte cultures co-cultured with iMGLs, stained for MAP2 and GFAP (left) and MAP2 and IBA1 (right) **(B)** UMAP embedding of scRNA-seq data from ReN neuron–astrocyte–iMGL co-cultures showing separation of mature neurons, astrocytes/immature neurons, and microglia. **(C)** Dot plot showing expression of canonical cell-type marker genes across annotated cell populations; dot size indicates the percentage of expressing cells and color denotes scaled average expression. **(D)** UMAP of microglial subclustering identifying tMG populations, annotated based on marker gene expression and functional signatures. **(E)** Heatmap of scaled expression (z-score) of selected marker genes across microglial clusters **(F)** Quantification of microglial cluster proportions across experimental conditions (WT_CTRL, WT_AD, KO_CTRL, KO_AD). Bars represent mean ± s.e.m. **(G)** Pathway enrichment analysis of selected microglial clusters (KEGG and Reactome). **(H)** Quantification of CD9 microglial density across co-culture conditions. **(I)** ELISA measurements of secreted Aβ40, Aβ42, and Aβ42/40 ratio in conditioned media from AD co-cultures with WT or MEF2C-KO iMGLs. **(J)** UMAP of neuron–astrocyte subclustering revealing distinct astrocytic and neuronal populations. **(K)** Quantification of neuron–astrocyte clusters across experimental conditions. Bars represent mean ± s.e.m **(L)** Gene set enrichment analysis (GSEA) of astrocytic signatures across species, shown as normalized enrichment scores (NES) **(M)** Feature plots showing enrichment of disease-associated astrocyte (DAA) signatures in AD co-cultures with WT or KO iMGLs. In H and I points represent individual iMGL clones; paired measurements are connected. In F, H, I, and K p-values were calculated using linear mixed-effects models followed by post hoc pairwise comparisons using estimated marginal means (emmeans); exact p-values are shown.

By using a combination of cellular models with complete or partial loss of MEF2C, we observed transcriptional changes consistent with increased DLAM signatures. However, whether these effects are sensitive to AD-relevant conditions or, in turn, may modulate AD-related biomarkers remains unknown. To address this, we utilized a previously published model of ReN cells expressing autosomal-dominant AD mutations in *PSEN1* and *APP*, with WT ReN cells serving as controls^62,63^. This model contains a mixed population of neurons and astrocytes, which were overlaid with either WT or MEF2C-KO iMGLs and co-cultured for one week. Our experimental design comprises four groups defined by neuronal context (control vs AD) and iMGL genotype (WT vs KO): CTRL_WT, CTRL_KO, AD_WT, and AD_KO (Figure 9A). First, we performed scRNA-seq on all four groups. After data integration and clustering, we recovered three major cell types: microglia, astrocytes/immature neurons, and mature neurons (Figure 9B, Supplementary Figure 7A,B). The mature neuronal population was very small and nearly absent in AD cultures, likely due to the sensitivity of these cells to dissociation and barcoding procedures^63^. Astrocyte/immature neuron clusters expressed *GFAP, AQP4, SLC2A1*, as well as *TUBB3* and *MAP2*, but lacked synaptic markers or markers of mature neuronal subtypes, such as *GAD65, GAD67, VGLUT*, or *TH* (Figure 9C, Supplementary Figure 7A). To determine whether CTRL or AD neurons influenced MEF2C-KO iMGLs, we subsetted microglia (cluster “Microglia”) and performed reclustering. This analysis identified 11 distinct microglial clusters, including DLAM-like clusters tMG_0 and tMG_4, a proliferative cluster tMG_6, and an MHC-II expressing cluster tMG_1 (Figure 9D). Several proinflammatory clusters were also identified (tMG_2, tMG_9, tMG_10), enriched for genes such as *CCL2, TNF, CCL20*, and *NFKB* (Figure 9D). Pathway analysis confirmed that tMG_0 and tMG_4 represent DLAM clusters, with enrichment for lysosomal, efferocytic, and cholesterol metabolism pathways (Figure 9G). We observed minimal differences in cluster proportions between CTRL_WT and AD_WT (Figure 9F). In contrast, MEF2C-KO iMGLs exhibited the most pronounced changes, particularly in DLAM-like clusters. Comparison of the four experimental groups revealed an increased proportion of tMG_0 in CTRL_KO (~11%) (tMG_0 KO_CTRL vs WT_CTRL: 0.10875 [−.0042, 0.2217] p=0.059) and an increased proportion of tMG_4 in AD_KO (~8%) (tMG_4 KO_AD vs WT_AD: 0.0641 [0.0225, 0.1057] p=0.003). Cluster projection analysis mapped tMG_0 to the iMG_0 main DLAM cluster, suggesting that its increased proportion in CTRL_KO reflects the phenotype observed in KO monocultures (Supplementary Figure 7C). Interestingly, in the AD_KO group, tMG_0 was not increased as compared to AD_WT (tMG_0 KO_AD vs WT_AD: 0.0048 [−.0852, 0.0949] p=0.9979) instead, tMG_4 was expanded (Figure 9F). Pathway analysis of tMG_4 marker genes implicated lysosome, neutrophil degranulation, immune system, and plasma lipoprotein clearance pathways (Figure 9G). Notably, CD9, a well-established human DAM and LAM marker^17,37^, was among the cluster markers for tMG_4 (Figure 9E, Supplementary Figure 7E). Immunofluorescence confirmed a statistically non-significant increase of CD9 expression in CTRL_KO and AD_KO compared to CTRL_WT and AD_WT respectively (Integrated density: CTRL_KO vs CTRL_WT: 6,073 [−1,744, 93,890] p=0.997; AD_KO vs AD_CTRL: 74,512 [−3,342, 173,367] p=0.149) (Figure 9H). However, interaction analysis showed a positive interaction effect between iMGL and ReN cell genotypes, indicating that the effect of iMGL genotype was enhanced in AD ReN cells (integrated density: 68,439 p=0.053). Interestingly, unpublished data from a CRISPR screen demonstrated that MEF2C-KD in iMGLs lead to higher expression of CD9^64^. To evaluate the impact of KO iMGLs on AD-related biomarkers, we measured Aβ40 and Aβ42 levels. Because control cultures exhibited very low Aβ levels, this analysis focused on the AD groups. The level of Aβ40 was non-statistically increased in AD_KO as compared to AD_WT supernatants (0.402 [−.509, 1.31] p=0.313), while the level of Aβ42 was comparable between these two groups (−0.348 [−1.41, −0.717] p=0.444). Consequently the Aβ42/40 ratio, a key AD biomarker, was reduced in AD_KO compared to AD_WT supernatants (0.438 [0.175, 0.701] p=0.015), suggesting loss of MEF2C in iMGLs may have a beneficial effect by modulating the ratio of Aβ species (Figure 9I). Finally, we examined the effects of KO iMGLs on adjacent astrocytes and neurons. Subsetting and reclustering astrocytes/immature neurons revealed several distinct clusters (Figure 9J). Changes in the proportions of these clusters were primarily driven by the cellular origin (CTRL vs AD) and were not affected by the presence of KO iMGLs, as shown by comparable proportions between KO_CTRL and WT_CTRL, as well as between KO_AD and WT_AD (Figure 9K, statistical details in Supplementary File 1). Given that microglia are known to modulate astrocyte activation and reactivity^65–67^, we next examined whether MEF2C-KO iMGLs alter astrocytic reactive states using gene signatures derived from mouse and human studies^67–71^ (Supplementary Data 7). We first performed pseudobulk differential expression analysis of neuron–astrocyte clusters, adjusting for clone and batch effects (Supplementary Data 7), followed by gene set enrichment analysis (GSEA). This analysis revealed a consistent reduction in multiple reactive astrocyte signatures, including disease-associated astrocytes (DAA) and A1 astrocytes identified in mouse studies, as well as human reactive astrocyte programs (Figure 9L,M). In contrast, astrocytes cocultured with MEF2C-KO iMGLs exhibited increased enrichment of protective A2 astrocyte signatures compared with those cocultured with WT iMGLs (Figure 9L and Supplementary Figure 7E,F). Collectively, these findings indicate that MEF2C-KO iMGLs exert beneficial effects on AD-related phenotypes by modulating DLAM-like microglial states, reducing toxic Aβ42/40 ratios, and promoting homeostatic and protective astrocyte transcriptional programs.

## Discussion

Here, we demonstrate that the AD–associated transcription factor MEF2C plays a central role in regulating macrophage molecular and cellular states. Genetic inactivation of MEF2C in human iMGLs induced robust transcriptional and functional DLAM responses, particularly those involved in lysosomal activity and cholesterol clearance. These findings were validated across four independent human iMGL lines, in THP-1 macrophages following partial MEF2C reduction, and in mouse microglia lacking Mef2c. We further show that MEF2C loss has broad effects at AD risk loci, altering the expression of genetically prioritized AD risk genes. Importantly, in a triculture model containing AD neurons and astrocytes, MEF2C-deficient iMGLs reduced the Aβ42/40 ratio, decreased signatures of reactive astrocytes, and increased expression of CD9, a conserved and well-established DLAM marker. Consistent with these findings, a recent study identified MEF2C among transcription factors whose reduction increases CD9 expression, further supporting MEF2C as a key driver of DLAM responses.

### Loss of MEF2C in iMGLs as a DLAM in vitro model

Given the central role of DLAM states in modulating microglial clearance pathways, establishing robust *in vitro* models of DLAM is essential for screening genetic and pharmacologic modifiers of this response. We and others have contributed to the development of several such models, which currently include stimulation with lipid-rich phagocytic substrates^43^, cytokine cocktails^46^, anti-tumor compounds^13^, HDAC inhibitors^72^, and transcription factor perturbations^20,43^. In this study, we add MEF2C inactivation or partial reduction as a new and physiologically relevant DLAM-inducing paradigm. Together with previously described perturbations—such as BHLHE40/41 loss or reduction^20^ and MITF overexpression^43^—MEF2C reduction provides another example of how modulating the levels of a single DLAM transcription factor can polarize microglia toward DLAM-like transcriptional and functional states. Importantly, we hypothesize that MEF2C may lie upstream of other DLAM TFs, based on two lines of evidence: (i) MEF2C binds the promoters of many transcription factors previously identified as DLAM regulators, and (ii) MEF2C-KO iMGLs exhibit motif enrichment for TF families known to drive DLAM phenotypes, including CEBP, MIT/TFE, and LXR. The MEF2C-deficient iMGL model appears to be among the most comprehensively characterized DLAM models to date, with congruent alterations observed at multiple regulatory layers. These include a clear transcriptional reprogramming toward DLAM signatures, shifts in DLAM state composition, widespread chromatin accessibility changes, and functional enrichment of lysosomal clearance and cholesterol efflux pathways.

### MEF2C and microglial identity

MEF2C is highly expressed in microglia and has been proposed to play a central role in establishing the microglial chromatin landscape^14^. Nevertheless, the generation of microglia lacking MEF2C is feasible and has been reported in both mouse and human systems^29–31^. These studies consistently describe MEF2C-deficient microglia as exhibiting a more amoeboid and less ramified morphology, yet they did not detect substantial loss of core microglial identity markers^29,31^. Our findings are consistent with these observations. We observed unchanged or modestly increased expression of canonical microglial markers, including TMEM119, P2RY12, and CD45, at the protein level. Although MEF2C has been proposed to regulate the CX3CR1^high^ microglial population during transcription factor–driven iMGL differentiation^28^ neither our study nor previous reports^31^ detected a substantial reduction in CX3CR1 protein levels, despite a reproducible decrease at the transcriptional level. Interestingly, loss of MEF2C was accompanied by increased expression of BAM markers. This shift was validated at the transcriptomic level in an independent iMGL line from^31^ as well as in mouse microglia lacking Mef2c^29^, suggesting that this phenomenon is conserved across species and experimental systems. Importantly, this BAM-like shift was observed only following complete genetic inactivation of MEF2C and was not recapitulated in THP-1 macrophages with partial MEF2C reduction. This suggests that full loss of MEF2C may be required to unmask this phenotype, or alternatively, that THP-1 macrophages lack the plasticity necessary to further polarize toward a BAM-like state. Importantly, the induction of BAM-associated markers may partially explain the enhanced clearance programs observed in MEF2C-deficient cells, as BAMs are known to be highly phagocytic and actively engaged in cellular debris clearance/efferocytosis^73^. The pronounced co-expression of BAM and microglial markers may be explained by several non-mutually exclusive mechanisms. First, co-activation of DAM- and BAM-associated transcriptional programs may occur only in *in vitro* models, as previously observed in iMGL exposed to phagocytic substrates, where both programs are induced concurrently. Such co-expression of DAM and BAM is not observed in human DAM *in vivo*. Second, WTC11-derived iMGL may resemble choroid plexus–associated macrophages (Kolmer cells or CP-BAM), which are frequently misclassified due to simultaneous expression of BAM and microglial markers and reduced homeostatic signatures^48,74,75^. Finally, iMGL may reflect an early fetal microglial state (~8 weeks gestation), during which BAM-associated markers are transiently expressed and subsequently downregulated upon entry into the brain parenchyma^76,77^. Together, these findings suggest that MEF2C does not abolish microglial identity per se, but instead constrains alternative myeloid programs that become evident upon its loss.

### MEF2C and inflammation

The effects of MEF2C loss or partial loss on the expression and secretion of pro-inflammatory cytokines remain arguable across model systems. The initial mouse study reported that transient MEF2C reduction in primary microglia exaggerated the secretion of TNFα, IL-1β, IL-1α, and IL-6 following acute LPS stimulation^29^. Consistent with this, MEF2C knockout in human microglia similarly increased expression of several inflammatory markers upon LPS exposure^30^. More recently, a study in iMGLs demonstrated that MEF2C-deficient cells secreted elevated levels of IL-1β, IL-6, IL-1α, and TNF even at baseline—an unexpected observation given the minimal transcriptional upregulation of these cytokines and the negative enrichment of pro-inflammatory myeloid signatures observed in the EC11 iPSC line with MEF2C loss^31^. A key consideration is that all of these studies employed acute, high-intensity inflammatory stimuli, including high-dose LPS, IFNβ, or TNFα. In contrast, our study utilized a milder LPS concentration and extended stimulation window (24 hours). This approach may better model adaptation to inflammatory cues rather than the immediate early response, potentially explaining the divergence between our findings and prior reports. Notably, a mouse model of MEF2C haploinsufficiency (MCHS), in which Mef2c heterozygosity was restricted to microglia, also did not show exaggerated inflammatory activation, supporting the idea that MEF2C loss does not universally sensitize microglia to inflammatory stimuli^78^. Furthermore, studies of other immune lineages suggest that MEF2C deficiency does not inherently promote hyperinflammation. Natural killer (NK) cells from MEF2C heterozygous mice or MCHS patients exhibited no increase in inflammatory responses; instead, when stimulated with IL-12 or IL-18, MEF2C-deficient NK cells produced less IFNγ than their wild-type counterparts^79^. Together, these observations suggest that MEF2C’s role in regulating inflammatory signaling is highly context-dependent, varying with stimulus strength, timing, and cell lineage, and may involve post-transcriptional or non-canonical mechanisms rather than direct transcriptional control of cytokine genes.

### MEF2C as a modulator of AD risk

Analysis of MEF2C-regulated epigenetic regions integrated with fine-mapping prioritized candidate variants predicted to be sensitive to MEF2C downregulation, whether through genetic inactivation or the physiological decline in MEF2C expression observed during aging. Linking these high-confidence variants to their putative target genes using Hi-C chromatin interaction data revealed enrichment for genes involved in lysosomal biology (*BIN1, INPP5D, CTSB, PICALM, SORL1, PLCG2, GRN*) and lipid metabolism (*TREM2, ABCA7, APOE*). These pathways align closely with the transcriptional and functional phenotypes observed across human iMGL, mouse microglia, and THP-1 macrophages, where MEF2C loss or reduction resulted in enhanced lysosomal activity and increased cholesterol handling. Consistently, putative myeloid AD risk genes nominated by our group^7^ were significantly enriched within a major DLAM cluster (iMG_0) characterized by lysosomal processing and efferocytosis - a biological hub strongly implicated by AD genetics^3,4,80^. Notably, this cluster exhibited the greatest sensitivity to MEF2C genetic inactivation and showed the largest shift in abundance between KO and WT conditions, underscoring its functional dependence on MEF2C activity. Furthermore, pathway analysis of putative myeloid AD risk genes intersecting with functional MEF2C targets demonstrated significant enrichment for amyloid clearance and processing pathways, linking the MEF2C regulatory network directly to microglial clearance functions central to AD pathogenesis.

To functionally assess how MEF2C loss modulates AD-relevant phenotypes in microglia, we employed a human 3D triculture model. In this system, MEF2C knockout iMGLs produced beneficial outcomes, including an increased proportion of DLAM, a reduced Aβ42/40 ratio, and decreased signatures of reactive astrocytes. Previous studies examining neuronal MEF2C have clearly demonstrated that loss of MEF2C in neurons is detrimental and associated with rapid cognitive decline, whereas higher MEF2C expression in excitatory neurons correlates with preserved cognitive function^22,23,81^. Consistently, MEF2C haploinsufficiency has been linked to autism spectrum disorder, where microdeletions leading to MEF2C downregulation contribute to neurodevelopmental pathology^82,83^. A recent study reporting a highly similar functional phenotype in MEF2C-deficient iMGLs (EC11 line) linked enhanced lysosomal activation to microglial hyperactivation and synapse loss^31^. This work suggests that chronic loss of MEF2C from development onward may be detrimental, as sustained overactivation and reduced plasticity can impair synaptic pruning, brain wiring, and ultimately behavior. In contrast, in the context of AD, a timely and context-dependent reduction of MEF2C may promote a DLAM response that facilitates amyloid clearance. Notably, understanding the consequences of MEF2C downregulation is particularly important in aging, as MEF2C is strongly downregulated in aged microglia, potentially as an adaptive mechanism to induce DLAM-like phenotypes.

### Limitation of the study

This study provides a comprehensive analysis of the role of MEF2C in human iMGLs at baseline, following stimulation with myelin fragments, under inflammatory conditions, and in the context of co-culture with AD-relevant neurons and astrocytes. However, several important aspects require further investigation. One limitation of this work is the limited assessment of biological sex as a variable. Most experiments were performed using male-derived lines, including WTC11- and EC11-derived iMGLs, THP-1 macrophages, and mouse datasets that were generated exclusively from males. To partially address this limitation, we performed transient MEF2C knockdown using siRNA in two female iPSC lines and observed transcriptional and protein-level phenotypes consistent with DLAM induction. Nevertheless, additional studies will be required to fully characterize sex-specific differences in MEF2C function and downstream microglial responses. A second limitation relates to the temporal dynamics of MEF2C regulation. While it is an intriguing hypothesis that a transient and precisely timed reduction of MEF2C may be beneficial by promoting a DLAM response when needed, we did not directly address timing effects in this study. This was largely due to the lack of an inducible CRISPR system that would allow controlled on–off modulation of MEF2C expression. Ongoing follow-up studies are aimed at addressing this limitation *in vivo*, particularly in the context of amyloid deposition. Finally, to fully understand the role of MEF2C in AD pathology, incorporation of experimental systems that explicitly model disease-relevant protein aggregates would be informative. In particular, assessing the impact of MEF2C-mediated DLAM induction in the presence of amyloid pathology, tau pathology, or ideally both, will be essential to determine whether enhanced DLAM responses are uniformly protective or context dependent. Perhaps more importantly, given our findings linking MEF2C to cellular debris clearance/efferocytosis, it will be critical to assess the impact of MEF2C-mediated DLAM induction in the context of models of neurodegeneration or demyelination, as well as during developmental stages in which synaptic pruning is at its peak.

Altogether, these data support a model in which MEF2C acts as a transcriptional gatekeeper of microglial lysosomal and lipid clearance programs which have been strongly implicated in the etiology of AD by both genetic and experimental evidence, as also shown in this study. Age-related decline or genetic perturbation of MEF2C may therefore shift microglia toward a high lysosomal, lipid clearance state. By integrating genetic risk, epigenetic regulation, and functional phenotypes, our findings position MEF2C as a central node linking aging-associated transcriptional remodeling to microglial mechanisms underlying AD susceptibility.

## Methods

### Bulk RNAseq

RNA from human THP-1 macrophages and human iMGLs was extracted using the RNeasy Plus Mini kit (Qiagen, 74136) following manufacturer’s instructions. mRNA quantity was measured using Nanodrop 8000 (Thermo Fisher Scientific). RNA was submitted to Azenta (New Jersey, NJ, USA) for QC, library preparation, and next-generation sequencing. Samples passed quality control with Qubit and BioAnalyzer showing RIN > 9.0. Libraries were prepared using TruSeq RNA Sample Prep Kit v2 and paired-end sequenced using Novaseq X at a read length of 150bp to obtain 20–30M mapped fragments per sample. Sequenced reads were processed via nf-core/rnaseq pipeline^85^ (doi: 10.5281/zenodo.1400710). Differential gene expression analysis (DGEA) was performed using a linear mixed model implemented in DREAM (differential expression for repeated measures, *variancePartition* R package v1.23.1 and R v4.3.3^86^). For iMGL datasets, we modeled the interaction between genotype and treatment, including differentiation batch and clone as random effects. For THP-1 macrophages, we examined the effect of genotype while adjusting for differentiation/transfection batch as a random effect. In addition, we reanalyzed published RNA-seq datasets from Mef2c-deficient mouse microglia reported by Deczkowska *et al*.^29^ (GSE98401), as well as an independent human iMGL line (EC11) lacking MEF2C generated by Nguyen *et al*.^31^ (GSE306993). Genes with an adjusted false discovery rate (FDR) < 0.05 were considered differentially expressed. To identify pathways enriched in MEF2C knockout (KO) or knockdown (KD) macrophages, we performed Gene Set Enrichment Analysis (GSEA). Ranked gene lists were generated from differential expression results by ordering genes according to the signed Z statistic. GSEA was conducted using the *fgsea* package (v1.28.0). Ranked lists were tested for enrichment against gene sets from the Molecular Signatures Database (MSigDB v7.5.1; Broad Institute), including *c2.cp.all.v2023.2.Hs.symbols.gmt* for human datasets and *m2.all.v2025.1.Mm.symbols.gmt* for mouse datasets, as well as curated myeloid gene sets compiled from published studies (see details in Supplementary Data 1 for human and Supplementary Data 4 for mouse). Results from DGEA and GSEA are provided in Supplementary Data 2 (iMGLs), Supplementary Data 3 (THP-1 macrophages), and Supplementary Data 4 (mouse microglia). Signatures extracted from mouse datasets were lifted to human orthologs using the Orthology search tool in gProfiler^87^. Enrichment scores were normalized by geneset size to generate normalized enrichment scores (NES) according to the standard protocol^88^.

### Single-cell RNAseq library preparation

Cells were first washed with PBS, then lifted with Accutase (StemcellTechnologies. cat.07920) for 10 min at 37°C. Cells were spun at 400×g for 5 min and resuspended in 1.0% PBS/bovine serum albumin (BSA). Cells were filtered through 40μm cell strainer to exclude cell clumps. Trypan blue was used to assess cell number and viability (typically > 90%) using an automated cell counter Countess (Thermo Fisher Scientific). Single cells were partitioned into PIPs (Fluent Biosciences, Watertown, MA, USA; PIPseq T20 3’ Single Cell RNA Kit v4.0 kit). Briefly, 40,000 live cells were added to a tube containing template beads thawed on ice, pipetted gently to mix, and then immediately vortexed for 3 minutes and processed further according to manufacturer’s instructions. Stable PIP emulsions containing captured mRNA after cell lysis were processed within 72h followed by cDNA generation, amplifications, cDNA purification and library preparation. Sequencing was performed by Azenta (New Jersey, NJ, USA) on a NovaSeq X sequencer with 50,000 reads per cell reaching 2 billions reads per sample.

### Single cell RNAseq data analysis

Raw base call files from the sequencer were demultiplexed into FASTQ files using PIPseeker v3.2(Fluent Biosciences). FASTQ files were aligned to the human reference genome (GRCh38) followed by filtering, barcode counting and UMI counting to generate a feature-barcode matrix per sample. For iMGLs we selected Sensitivity 3, for triculture we selected Sensitivity 4. Quality control, normalization, clustering and marker gene identification were performed with Seurat v5.0.2^89^. Briefly, cells were removed if they were expressing fewer than 100 genes, less than 500 UMIs and greater than 95% of total UMIs, or if greater than 10% of reads mapped to the mitochondrial genome. Raw count data was normalized using SCTransform v0.4.1^90^, with regression for mitochondrial mapping percentage. Principal components analysis (PCA) was performed to determine the top most variable genes and generate PCs. Samples were integrated using *harmony* v1.2.0^91^ to account for batch differences. Clustering and data reduction were performed in Seurat using UMAP for visualization. The number of cells per batch were randomly downsampled to the smallest treatment condition to ensure that differences in proportions were not due to variable cell counts. Marker genes for each cluster were identified using Seurat *FindMarkers*. Cluster annotations were determined using a combination of approaches, (1) high expression of established marker genes from the literature; (2) enrichment of myeloid gene expression signatures from published datasets (Supplementary Data 1) using hypergeometric overlap; (3) enrichment of biological pathways in the expressed genes to determine functional significance. We used the *propeller* method via the *speckle* package (v0.99.7) in R to identify statistically significant differences in cell proportions between treatment conditions for each cluster, with adjustment for clone and using adj.p < 0.05 as a cutoff. For comparison of clusters identified in this study with iMGL clusters we used the *scmap* package (v1.20.0)^47^. Briefly, Seurat objects were converted to SingleCellExperiment objects (*SingleCellExperiment* package, v1.20.1). Projection of query (this study) and reference clusters^43^ was performed using scmap-cluster projection with a default feature selection (n_feature = 500). Enrichment of transcriptional programs was tested using the *AddModuleScore* function in Seurat. For association of ROSMAP traits with iMGL clusters, we utilized previously published genes positively and negatively associated with AD traits^13^ (see “Examining association of ROSMAP traits with clusters” and Supplementary Table 3). We first filtered positively (adj.p < 0.05), log2FC > 0) and negatively (adj.p < 0.05, log2FC < 0) associated genes with each AD trait. Then we performed hypergeometric overlap between either positively or negatively associated genes with cluster marker genes (i.e. gene upregulated in each mac cluster, provided in Supplementary Data 6). The hypergeometric overlap p values were FDR-corrected. The hypergeometric overlap between cluster marker gene and myeloid gene sets was performed using *hypeR* package (v3.2.2)^92^. FDR-corrected p values are listed in Supplementary Data 6.

### FreshMG reanalysis

Raw FASTQ files and meta data from Lee et al.,^93^ were downloaded from the Synapse portal (syn52795287). Fastq files were processed using CellRanger v9.0 (10x Genomics). CellBender^94^ and scDblFinder^95^ were used to remove ambient RNA and doublets, respectively. Microglia were identified and analyzed using the Seurat package (v4.3), with normalization performed using SCTransform^90^ and batch correction using Harmony^91^ The dreamlet package^96^ was used to identify genes associated with age, employing the following model: 1 + scale(Age) + Sex + Ancestry + Sequencing + Source + scale(PMI). Variance partition and canonical correlation analyses were performed using the variancePartition package (v1.2) to determine appropriate covariates prior to model fitting. Pearson correlation analysis was used to assess the relationship between MEF2C expression and age (corresponding to Figure 1E).

#### ATAC-seq

Cells were harvested using Accutase (Stemcell Technologies, cat.07920) and washed once with PBS. Cells were resuspended in Synth-A-Freeze freezing medium (Thermo Fisher Scientific, cat.A1254201) and cryopreserved at −80°C. DNA library preparation and sequencing were performed by Azenta (New Jersey, NJ, USA). Raw ATAC-seq data from WTC11-derived iMGLs generated in this study, together with EC11-derived iMGLs generated by Nguyen *et al*.^31^ were processed using the nf-core/atacseq pipeline^85^ (doi: 10.5281/zenodo.2634132) with default parameters. Differentially accessible regions were identified using *DiffBind* (v3.16.0) using default parameters and subsequently annotated using *ChIPseeker* (v1.42.0). All processed datasets and peak annotations are provided in Supplementary Data 5. TF motif analysis was performed using HOMER’s motif analysis using differentially accessible regions (*findMotifsGenome.pl*) including known default motifs and *de novo* motifs was used; known motifs were reported in this manuscript. Background sequences were derived from WT genomic sequences of the same lengths as analyzed peaks.

### ChIPseq

H3K27ac and MEF2C ChIP–seq data were generated by Nguyen *et al*.^31^. Raw sequencing data were downloaded from GEO (GSE306993) and processed using the nf-core/chipseq pipeline with default parameters^85^ (10.5281/zenodo.3240506). For MEF2C ChIP–seq, each immunoprecipitated sample was normalized to its corresponding input (no-antibody) control. Statistically significant peaks were identified from the MACS3 output. MEF2C binding sites were defined as peaks present in both wild-type (WT) iMGL replicates and absent in the MEF2C-KO iMGL sample. For H3K27ac ChIP–seq, immunoprecipitated samples were similarly normalized to their respective input controls. Differentially acetylated regions were identified using *DiffBind* (v3.16.0), followed by genomic annotation with *ChIPseeker* (v1.42.0). All processed datasets and peak annotations are provided in Supplementary Data 5.

### RT-qPCR

Cell pellets were used for mRNA extraction using the RNeasy Plus Mini kit (Qiagen, 74136) following manufacturer’s instructions. mRNA quantity was measured using Nanodrop 8000 (Thermo Fisher Scientific) and reverse transcription reaction was performed with 500 ng of RNA using High-Capacity RNA-to-cDNA kit (Thermo Fisher #4387406). 5 ng cDNA was used in the qPCR reaction with Power SYBR Green Master Mix (Applied Biosystems, 4368706) run using QuantStudio 7 Flex Real-Time PCR System (Thermo Fisher Scientific). Primers were designed using the IDT software and are listed in Supplementary Data 8. Ct values were averaged from two technical replicates for each gene, *GAPDH* Ct values were used for normalization. Gene expression levels were quantified using the 2^−ddCt^ method relative to control. Log2 fold change data for each marker were analyzed and plotted.

### Flow cytometry

Cells were detached using Accutase (Stemcell Technologies, cat. 07920) for 10 min at 37°C using. Cells were spun at 400g for 5 min and resuspended in 1.0% PBS/bovine serum albumin (BSA).

For single marker experiment cells were pre-stained with the following dyes/antibodies: Live/Dead Fixable Violet Dead Cell Stain kit (Life Technologies, cat. L34955) to exclude dead cells, CD11b-FITC (BioLegend, cat. 101206) and one of the DAM markers CD11c-APC 1:100 (BioLegend, cat. 301614), CD63-APC 1:100 (BioLegend, cat. 353007), TREM2-APC 1:50 (R&D Systems, cat. FAB17291A). Only one DAM marker was used at a time. Cells were washed twice in 1.0% PBS/BSA buffer and analyzed by Attune flow cytometer (Thermo Fisher Scientific). Gates were set up based on fluorescence minus one (FMO) controls. Data were analyzed using FCS Express 7 (De Novo Software). Percentage of Live/Dead^Neg^/CD11b^Pos^/DAM^Pos^ for each marker was exported and analyzed.

For spectral flow cytometry, two antibody panels were generated: an identity panel that includes following markers: CD206-BUV395 (1:25, BD Biosciences, cat. 740309), CD11c-BUV661 (1:50, BD Biosciences, cat. 612967), CD14-BV421 (1:100, BioLegend, cat. 367144), HLA-DR-BV605 (1:100, BioLegend, cat. 307640), CD11b-BV785 (1:100, BioLegend, cat. 101243), CD206-FITC (1:100, BioLegend, cat. 321104), TMEM119-PE (1:100, BioLegend, cat. 853316), P2RY12-PE-Dazzle594 (1:150, BioLegend, cat. 392112), CD163-PECy5 (1:100, BioLegend, cat. 333644), CX3CR1-PECy7 (1:100, BioLegend, cat. 341612), CD64-APC (1:100, BioLegend, cat. 305014), CD14-Spark NIR 685 (1:100,BioLegend, cat. 367150), and CD45-APC-Cy7 (1:100, Tonbo Biosciences, cat. 25–049-T100), and a DAM panel that was comprised of following markers: HLA-DR-BV605 (1:100, BioLegend, cat. 307640), CD33-BV711 (1:50, BioLegend, cat. 303423), CD11b-BV785 (1:100, BioLegend, cat. 101243), CD11c-BB515 (1:100, BD, cat. 564490), Clec7a-PerCP-eFluor 710 (1:100, Thermo Fisher Scientific, cat. 46-9856-42), CD44-PerCP-Fire 806 (1:100, BioLegend, cat. 103082), GPNMB-PE (1:100, Thermo Fisher Scientific, cat. 12-9838-42), P2RY12-PE-Dazzle594 (1:150, BioLegend, cat. 392112), CD36-PE-Fire 640 (1:100, BioLegend, cat. 336246), CX3CR1-PE-Cy7 (1:100, BioLegend, cat. 341612), TREM2-APC (1:50, R&D Systems, cat. FAB17291A), CD9-Spark Red 718 (1:150, BioLegend, cat. 312129), and CD45-APC-Cy7 (1:100, Tonbo Biosciences, cat. 25-049-T100). After a PBS wash, cells were first stained with Live/Dead Fixable Violet Dead Cell Stain kit (see above) together with human FC block (BD, cat. BDB564220) in D-PBS for 30 min at 4°C. Cells were washed twice in PBS followed by resuspension in antibody cocktail of either identity panel or DAM panel. The staining buffer was composed with 50% FACS buffer (D-PBS + 0.5% BSA) and 50% of Brilliant Stain buffer (BD, cat. 563794) and antibody cocktails. Cells were stained for 30 min at 4°C, followed by two washes in FACS buffer and fixation in 4% paraformaldehyde (Electron Microscopy Science, cat. 50-980-487) for 10 min at room temperature. Next, cells were washed twice in PBS, resuspended in FACS buffer and analyzed within 72h using a 5-laser spectral flow cytometer Aurora (Cytek Bioscience). Reference controls were prepared with a combination of beads (Invitrogen, cat. 01-3333-42) and cells. Data were analyzed using FlowJo v10.8 software (BD Life Science). gMFI per each marker was analyzed and plotted.

#### CytoVI analysis:

High-dimensional flow cytometry data were analyzed using CytoVI, a deep generative model based on the scVI framework. Briefly, data were normalized and scaled prior to model training. CytoVI learned a low-dimensional latent representation while correcting for acquisition batch effects. Leiden clustering was performed on the latent space, and UMAP was used for two-dimensional visualization.

### ELISA

Conditioned media from mature iMGLs and THP-1 macrophages were collected at the same time points as RNA harvest. Secreted protein levels were quantified using ELISA kits for APOE (Invitrogen, cat. EHAPOE), IL-6 (Abcam, cat. ab170813), IL-1β (Abcam, cat. ab214025), TNFα (Abcam, cat. ab181421), and CCL2 (Abcam, cat. ab179886), according to the manufacturers’ instructions. For APOE and CCL2 measurements, supernatants were diluted 1:10 in sample diluent prior to analysis.

### Phagocytosis

Uptake of different bioparticles was measured by flow cytometry (FACS). The following bioparticles were used: human myelin fragments isolated from AD brain (20 μg/ml), 5 μg/ml zymosan particles (Thermo Fisher Scientific, cat. P35364), Carboxyl Fluorescent Polystyrene 1.0μm particles (CD Bioparticles cat. DCFG-L007) for 24h. Myelin fragments were isolated from human brain tissue (corpus callosum) as described previously^97^. Isolated myelin fragments were labeled with pHrodo dye (Thermo Fisher Scientific, cat. P36600) (10μg/ml) in PBS for 30 min in the dark at room temperature followed by two washes in PBS. After 24-hour incubation cells were collected with gentle pipetting and Accutase (Stemcell Technologies, cat. 07920), washed twice, resuspended in 1.0% PBS/bovine serum albumin (BSA) buffer and analyzed on an Attune flow cytometer (Thermo Fisher Scientific). Cells were pre-stained with Live/Dead Fixable Violet Dead Cell Stain kit (Life Technologies, cat. L34955) to exclude dead cells. Data were analyzed using FCS Express 7 (De Novo Software). Live/Dead^Neg^/pHrodo^+^ cells were used to quantify the phagocytic index: percentage of pHrodo^+^ cells gated population × geometric mean pHrodo intensity; and represented as phagocytic activity as previously described^5^. Gates were set up based on fluorescence minus one (FMO) controls.

### Scratch wound assay

The Incucyte Scratch Wound Assay (Sartorius) is a real-time and automated method for studying cell migration and wound healing. iMGLs (50,000 cells/well) were seeded in a 96-well tissue culture plate and differentiated to macrophages. A scratch wound was generated in the cell monolayer using a 96-pin WoundMaker tool. One wash was performed to eliminate cell debris. The plate was then placed into the Incucyte Live-Cell Imaging System, where images were captured every hour. The Incucyte software analyzed the images and enabled the quantification of relative wound density that measures the ratio between area covered by cells over initial area of the wound in each time point. We performed 3–5 technical replicates per differentiation. Relative wound density averaged per genotype is plotted. Area under curve (AUC) was calculated using trapezoid approximation and analysed.

### Cholesterol efflux assay

Cholesterol efflux was performed using a Cholesterol Efflux Fluorometric Assay kit (Biovision, K582–100) following the manufacturer’s instructions. For this assay, cells were seeded in a 96-well plate at 50,000 cells/well. Cells were labeled with a Labeling Reagent for 1h at 37°C followed by loading cells with an Equilibration Buffer. After overnight incubation, media containing Equilibration Buffer was aspirated and replaced with media containing cholesterol acceptor human HDL (40 μg/ml) for 5h at 37°C. At the end of the incubation, supernatants were transferred to flat bottom clear 96-well white polystyrene microplates (Greiner Bio-one, 655095). Adherent cell monolayers were lysed with Cell Lysis Buffer and incubated for 30 min at RT with gentle agitation followed by pipetting to disintegrate cells. Cell lysates were transferred into flat bottom clear 96-well white polystyrene microplates. Fluorescence intensity (Ex/Em=485/523nm) of supernatants and cell lysates was measured using a Varioskan LUX multimode microplate reader (Thermo Fisher Scientific, VL0000D0). Percentage of cholesterol efflux was quantified: %cholesterol efflux = Fluorescence intensity of supernatant / fluorescence intensity of supernatant plus fluorescence intensity of cell lysate × 100. Fluorescence intensity of supernatant (fluorescence_s) was analysed using fluorescence intensity of cell lysate (fluorescence_l) as a covariate. % of cholesterol efflux was plotted.

### Lysosomal assay

Cells were incubated with 1 μM LysoSensor-Green (Thermo Fisher Scientific, L7535) staining for 1 min at 37°C. To characterize hydrolytic capacity of lysosomes, cells were treated with 1μg/ml DQ Red BSA (Thermo Fisher Scientific) for 1h at 37°C. After collecting the cells, single-cell data were acquired using Attune flow cytometer (Thermo Fisher Scientific, cat.D12051) and analyzed using FCS Express 7 (De Novo Software). Gates were set up based on fluorescence minus one (FMO) controls. LysoSensor and DQ-BSA gMFI were analysed and plotted.

### Lipid droplets assay

Neutral lipid droplet (LD) quantification was performed using flow cytometry. Cells were collected and stained with 3.7 μM BODIPY 493/503 (Thermo Fisher Scientific, cat.D3922) for 30 minutes at room temperature (RT), protected from light. Single-cell data were acquired using Attune flow cytometer (Thermo Fisher Scientific) and analyzed using FCS Express 7 (De Novo Software). Gates were set up based on fluorescence minus one (FMO) controls. BODIPY gMFI was analysed and plotted.

### Western Blotting

Cells were lysed in RIPA buffer (Thermo Scientific, 89900) supplemented with Protease/Phosphatase Inhibitor Cocktail (Cell Signaling, 5872) following manufacturer’s instructions. Protein concentration was measured using BCA kit (Thermo Fisher Scientific, 23225) and equal quantities were used to prepare samples for western blotting. Samples were resolved by electrophoresis with Bolt 4–12% Bis-Tris Plus Gels (Invitrogen) in Bolt MES SDS running buffer (Invitrogen, B0002) and transferred using iBlot 2 nitrocellulose transfer stacks (Invitrogen). Membranes were blocked for 1 h and probed with antibodies: APOE 1:1000 (Millipore, AB947), ABCA1 1:1000 (Abcam, 018180), LAMP1 (D2D11) 1:1000 (Cell Signaling Technology 9091S), CTSB (D1C7Y) 1:1000 (Cell Signaling Technology, 31718), LGALS3 1:300 (R&D, AF1197-SP), GPNMB 1:200 (R&D, AF2550-SP), PLIN2 1:500 (Proteintech, 15294–1-AP), ACTIN 1:10,000 (Sigma-Aldrich, A5441) in 5% non-fat dry milk in PBS/0.1% Tween-20 buffer overnight at 4 °C. Secondary antibody staining 1:10000 was applied for 1 h at RT, visualized using WesternBright ECL HRP Substrate Kit (Advansta, K-12045), and measured using iBrigh imagining system (Applied Bioscience). Images were analyzed using ImageJ (NIH). Uncropped western blot images are pasted in Supplementary Figure 8.

### Generation of MEF2C knockout iPSC line

The WTC-11 donor human iPSC line (Coriell Institute for Medical Research, GM25256) was tested for genomic integrity at passage 36 using SNP-array technology (Global Diversity Array v1.0 BeadChip, Illumina). No detection of CNV larger than 1.5 Mb or AOH larger than 3 Mb were detected on somatic chromosomes. The typical WTC-11 deletion of 2.9 Mb on Yp11.2 was detected. This deletion is known to be present in the donor, from whom the cell line was derived^98^. CRISPR Cas9 mediated knockout cell pool of MEF2C in WTC11 were generated by EditCo Bio, Inc. (Redwood City, CA, USA).

To generate these cells, Ribonucleoproteins containing the Cas9 protein and synthetic chemically modified guide RNA produced by Synthego were electroporated into the cells using EditCo’s optimized protocol. Editing efficiency is assessed upon recovery, 48 hours post electroporation. Genomic DNA is extracted from a portion of the cells, PCR amplified and sequenced using Sanger sequencing or NGS. The resulting Sanger chromatograms are processed using EditCo’s Inference of CRISPR edits software (https://ice.editco.bio/#/) or NGS bioinformatics pipeline. WT pool was generated by mock transfection with an empty vector.

To create monoclonal cell populations, edited cell pools are seeded at 1 cell/well using a single cell printer into 96 or 384 well plates. All wells are imaged every 3 days to ensure expansion from a single-cell clone. Clonal populations are screened and identified using the genotyping strategy described above. Below are the edit information:

Gene Name: MEF2C; Transcript ID: ENST00000504921.7; Guide RNA sequence: GAGUUUGUCCGGCUCUCAUG and target exon 3 with a frameshift deletion −11 bp; Guide RNA cut location: chr5:88,804,628; Exon targeted: 3; PCR Primers: Forward (5’ → 3’: GGTGGAGTAAGAGTTGCGAT, Reverse (5’ → 3’): CTTTCTGAATGTGTTAGTGCCC; Sequencing primer: Forward, GC enhancer used: No

Four karyotypically normal clones per genotype were generated. iPSC-derived microglia (iMGLs) were routinely differentiated and analyzed in paired fashion, with one WT clone processed alongside one KO clone within the same differentiation batch. Clone pairing was assigned blindly prior to all experimental procedures, and paired clones were always analyzed together to control for batch effects. In figures presenting functional data from MEF2C-KO iMGLs, paired clones are indicated by grey connecting lines.

### iPSC-derived microglia

Human induced pluripotent stem cells (iPSCs) were maintained on Matrigel (BD Biosciences) in complete mTeSR Plus (STEMCELL Technologies, cat. 100–0276). iPSCs were passaged every 5–6 days using ReLeSR dissociation reagent (STEMCELL Technologies, cat. 05872) and used for hematopoietic stem cell differentiation with STEMdiff Hematopoietic kit (STEMCELL Technologies, cat. 05310) followed by differentiation to induced microglial-like cells (iMGLs) using a previously published protocol^99^. iPSC lines were confirmed to have a normal karyotype (KaryoStat assay, Thermo Fisher Scientific). iMGLs were maintained and fed with a microglial medium supplemented with three factors (100 ng/ml IL34, 50 ng/ml TGFβ, 25 ng/ml MCSF) for 25 days. On day 25, iMGLs were additionally supplemented with two factors (CX3CL1 and CD200, 50 ng/ml each) for an additional three days. Mature iMGLs (day 28) were used for all downstream experiments.

### iMGL with transient reduction of MEF2C

Four independent iPSC lines were used to transiently reduce MEF2C expression: F12455 (female, European ancestry), ADRC5 (female, European ancestry), KOLF2.1J (male, European ancestry), and WTC11 (male, East Asian ancestry). All lines were APOE ε3/ε3 and derived from neurologically normal, non-AD individuals. Mature iPSC-derived microglia (iMGLs; day 28 of differentiation) were transfected with 40nM MEF2C siRNA (Horizon Discovery, L-009455–00) or 40 nM non-targeting control siRNA pool (Horizon Discovery, D-001810-10-05). Cells were harvested 48 hours post-transfection for downstream RNA and protein extraction.

### THP-1 macrophages with transient reduction of MEF2C

THP-1 human monocytic leukemia line was differentiated to human THP-1 macrophages using phorbol 13-myristate 14-acetate. THP-1 monocytes were derived from peripheral blood from an acute monocytic leukemia patient (a 1 year old male of East Asian ancestry (Japan)). THP-1 monocytes were purchased from ATCC. The line was not authenticated using short tandem repeat profiling (STR) or any other method. We tested for the presence of AD mutations in THP-1 macrophages by using published WGS data from THP-1 https://www.ncbi.nlm.nih.gov/sra/SRX5466705. We screened for several protein coding mutations using Variant Quality Recalibration Score (VQSR) and we found no altered alleles. The list of variants we screened for can be found in Table S5 from^46^. Cells were tested monthly for mycoplasma and consistently tested negative.

THP-1 monocytes were cultured in RPMI medium supplemented with 10% FBS, 1x Penicillin Streptomycin (1,000U/ml Penicillin 1,000μg/ml Streptomycin) and 10mM HEPES. 25ng/ml of phorbol 12-myristate 13-acetate (PMA) was used to differentiate THP-1 monocytes to macrophages. After 3 days, PMA was removed and replaced with serum-free media (10% FBS was replaced with 1% BSA). Differentiated macrophages were transfected using Lipofectamine RNAiMAX (Thermo Fisher Scientific, LMRNA015) with 40nM *MEF2C* siRNA (Horizon Discovery L-009455–00), or 40nM of non-targeting control pool (Horizon Discovery D-001810-10-05). THP-1 macrophages rested up to 48 h (including transfection) in serum-free media (1% BSA supplementation) prior to collection. There was no additional supplementation of media with lipid that would affect the final results. Changes in MEF2C expression were confirmed at the mRNA level using RT-qPCR and at the protein level using western blotting. We performed 4–6 independent macrophage differentiation and transfection.

### Treatment with myelin, LPS and IFNγ

Mature iMGLs were treated with either myelin fragments (see Phagocytosis) at 20 μg/ml or lipopolysaccharide (LPS O55:B5, Sigma-Aldrich cat.L2880) at 50 ng/ml for 24 h prior to collection. Cells were subsequently washed with PBS and harvested for RNA extraction. Culture supernatants were collected and stored for downstream ELISA analyses. THP-1 macrophages were treated with LPS (50 ng/ml) or interferon-γ (IFNγ; R&D cat.285-IF) at 50 ng/ml for 24 h, after which cells were collected for RNA isolation.

### 3D triculture model

ReN cell VM human neuronal progenitor cells (NPCs) were obtained from the laboratories of Drs. Rudolph Tanzi and Doo Kim. Two types of cells were used: control cells expressing GFP and AD cells harboring mutations in APP (Swedish and London) and PSEN1 (ΔE9). AD cells expressed both GFP and mCherry. For maintenance, ReN NPCs were plated onto BD Matrigel–coated T75 flasks (Corning, cat.356231) and cultured in expansion/maintenance medium consisting of DMEM/F12 (Fisher Scientific, cat.10-565-042) supplemented with heparin (2 μg/mL; Stemcell Technologies, cat.07980), 2% B27 neural supplement (Thermo Fisher Scientific, cat.17504044), EGF (20 ng/mL; Sigma-Aldrich cat.E9644), bFGF (20 ng/mL; Reprocell cat.03–0002), and 1× penicillin/streptomycin (Sigma-Aldirch, cat.P4333). Media were changed every 72 h until cultures reached ~80% confluency. For 3D neuron/astrocyte differentiation, cells were dissociated with Accutase (Stemcell Technologies cat.07920), counted, and plated at 250,000 cells per well in 96-well plates (Greiner Bio-One, cat.655866) in expansion/maintenance medium supplemented with 10% cold matrigel. The following day, the medium was completely replaced with a differentiation medium consisting of a maintenance medium without EGF and bFGF. Media were refreshed by 50% every 3–4 days or more frequently if required. After four weeks of differentiation, mature neuronal/astrocytic cultures were overlaid with mature iPSC-derived microglia (iMGLs). A total of 50,000 mature iMGLs were added per well, and the neuronal/astrocyte medium was supplemented with 3F cytokines to support iMGL survival. Tricultures were maintained for one week prior to downstream analyses.

#### Single-cell RNA sequencing:

Tricultures were dissociated using the Papain Dissociation System (Worthington Biochemical, cat.LK003153) according to the manufacturer’s instructions. Dissociation was completed within 40 min, after which cells were immediately processed for partitioning using PIPseq (see scRNA-seq section).

#### Immunofluorescence:

Tricultures were gently washed with PBS and fixed in 4% paraformaldehyde (Electron Microscopy Sciences, cat.15710) for 15 min at room temperature. Cells were permeabilized with 0.1% Triton X-100 and 0.05% Tween-20 in PBS for 15 min, followed by blocking of nonspecific binding with 5% normal donkey serum (Jackson ImmunoResearch, cat.017-000-121). Primary antibodies (anti-CD9 mouse monoclonal (clone HI9a) (BioLegend, cat.312102) 1:100, anti-beta-III Tubulin (R&D System, cat.MAB1195) 1:500, anti-GFAP (GA5) mouse monoclonal antibody (Cell Signaling Technology, cat.3670S) 1:1000, anti-IBA1 rabbit polyclonal antibody (Thermo Scientific, cat.PA5–27436) 1:500) were applied in different combination overnight at 4°C. Cultures were then washed three times with PBS and incubated with fluorophore-conjugated secondary antibodies for 1.5 h at room temperature. After washing, samples were maintained in PBS and imaged using a Stellaris confocal microscope (Leica, USA). The following secondary antibodies were used: donkey anti-mouse Alexa Fluor-647 (Thermo Fisher Scientific, cat.SA000059) and donkey anti-rabbit Alexa Fluor-750 (Thermo Fisher Scientific, cat.SA000087).

#### Image quantification:

Twelve-bit images were acquired using a Stellaris confocal microscope (Leica Microsystems). To quantify CD9 expression, an IBA1-positive mask was generated to define individual microglial cells using Fiji software^100^. This mask was overlaid onto the CD9 channel, and raw fluorescence intensity was extracted as the integrated density (sum of pixel intensities) within the masked area for each cell.

### Rank-rank hypergeometric overlap (RRHO)

Transcriptional signatures were compared pairwise using the RRHO2 R package^101^. Z.std values or −log10(P-value) * sign(log2FC) metrics were used to generate ranked lists of genes for each transcriptional signature. RRHO2 was then used to visualize both concordant and discordant gene expression changes across each pair of signatures as rank-rank hypergeometric overlap (RRHO) heatmaps. The color temperature of each pixel in an RRHO heatmap represents the negative log10- transformed hypergeometric overlap test P-value of subsections of the two ranked gene lists, adjusted for multiple testing using the Benjamini-Hochberg correction method. Heatmaps generated using RRHO2 have top-right (both decreasing) and bottom-left (both increasing) quadrants, representing concordant gene expression changes, while the top-left and bottom-right quadrants represent discordant gene expression changes. The default step size and p representation method (hyper) were used.

### Fine-mapping with PolyFun + SuSie

Alzheimer’s disease (AD) GWAS summary statistics used in this study were obtained from the EBI GWAS Catalog (https://www.ebi.ac.uk/gwas/) and restricted to individuals of European ancestry (GCST90027158; 39,106 AD cases, 46,828 proxy cases, and 401,577 controls)^53^. Functionally informed fine-mapping of AD risk variants was performed using PolyFun (https://github.com/omerwe/polyfun)^59^. Fine-mapping incorporated microglial epigenomic annotations, including differentially accessible regions identified by ATAC-seq in WTC11- and EC11-derived^31^ iMGLs, differentially acetylated H3K27ac peaks from EC11 iMGLs^31^, and MEF2C ChIP-seq peaks^31^. Prior causal probabilities were estimated using functional annotations for approximately 19 million UK Biobank–imputed SNPs (minor allele frequency > 0.1%). These priors were integrated using an L2-regularized extension of partitioned linkage disequilibrium score regression (LDSC), leveraging precomputed UK Biobank LD scores. Functionally informed fine-mapping was then conducted using the sum of single effects (SuSiE) model.

### Statistical analysis

All MEF2C knockout (KO) iMGL data were generated from a single iPSC line (WTC11) using four independent clones per genotype; accordingly, each clone was treated as an independent biological unit. Each clone was differentiated at least twice, and multiple technical replicates were included in functional assays to account for technical variability. To appropriately model the hierarchical structure of the experimental design and fully leverage the generated data, we used linear mixed-effects models implemented in *lme4* (v1.1.37). In statistical models, clones and differentiation batches next with technical replicates were incorporated as a random effect. MEF2C knockdown (KD) iMGL data were generated from four independent iPSC lines including random effects for line, differentiation batch nested within line, and transfection well nested within batch. KD THP-1 macrophage data were generated from a single THP-1 line across multiple independent differentiations and transfections, which were treated as random effects. Post hoc comparisons were performed using *emmeans* (v1.11.2.8) to estimate contrasts, p values, effect sizes, and confidence intervals. Primary statistical results are reported in the main text and figure legends. Complete statistical outputs, including *lme4* model summaries (generated with *report*, v0.6.1) and *emmeans* results, are provided in Supplementary File 1. For full transparency, all raw data and associated metadata are available in the Source Data file.

## Supplementary Material

1

Supplementary Files

This is a list of supplementary files associated with this preprint. Click to download.

- SupplementaryFile1Descriptivestatistics.docx

## Data Availability

All aligned read counts, fastQ files, and metadata from bulk RNAseq, bulkATACseq and scRNAseq with genetic inactivation of MEF2C including non treated, myelin treated, LPS treated conditions and tricultures as well as THP-1 macrophages with reduced MEF2C expression have been deposited to the Gene Expression Omnibus are available under following accession numbers GSE323984 (iMGLs all conditions bulk), GSE324006 (iMGLs all conditions scRNAseq), GSE323982 (triculture scRNAseq), GSE323981 (bulk ATACseq). Previously reported data are available from Gene Expression Omnibus, including those from Nguyen et al., 2025^31^ GSE306993 (bulk RNAseq, H3K27ac ChIPseq, ATACseq, and MEF2C ChIPSeq, EC11 iMGLs), and Deczkowska et al., 2017^29^ GSE98401 (bulk RNAseq, mouse microglia). Source data are provided with the paper.

## Code availability

This manuscript does not report original code
