# Supplementary material for: MEF2C controls lysosomal and lipid clearance programs linked to Alzheimer’s disease risk in macrophages
Source: Res Sq. 2026 Apr 28:rs.3.rs-9164252. Preprint. [Version 1] doi: 10.21203/rs.3.rs-9164252/v1 (PMC13142631; doi:10.21203/rs.3.rs-9164252/v1)
Supplement: 1 [file NIHPPRS9164252V1-supplement-1.pdf]

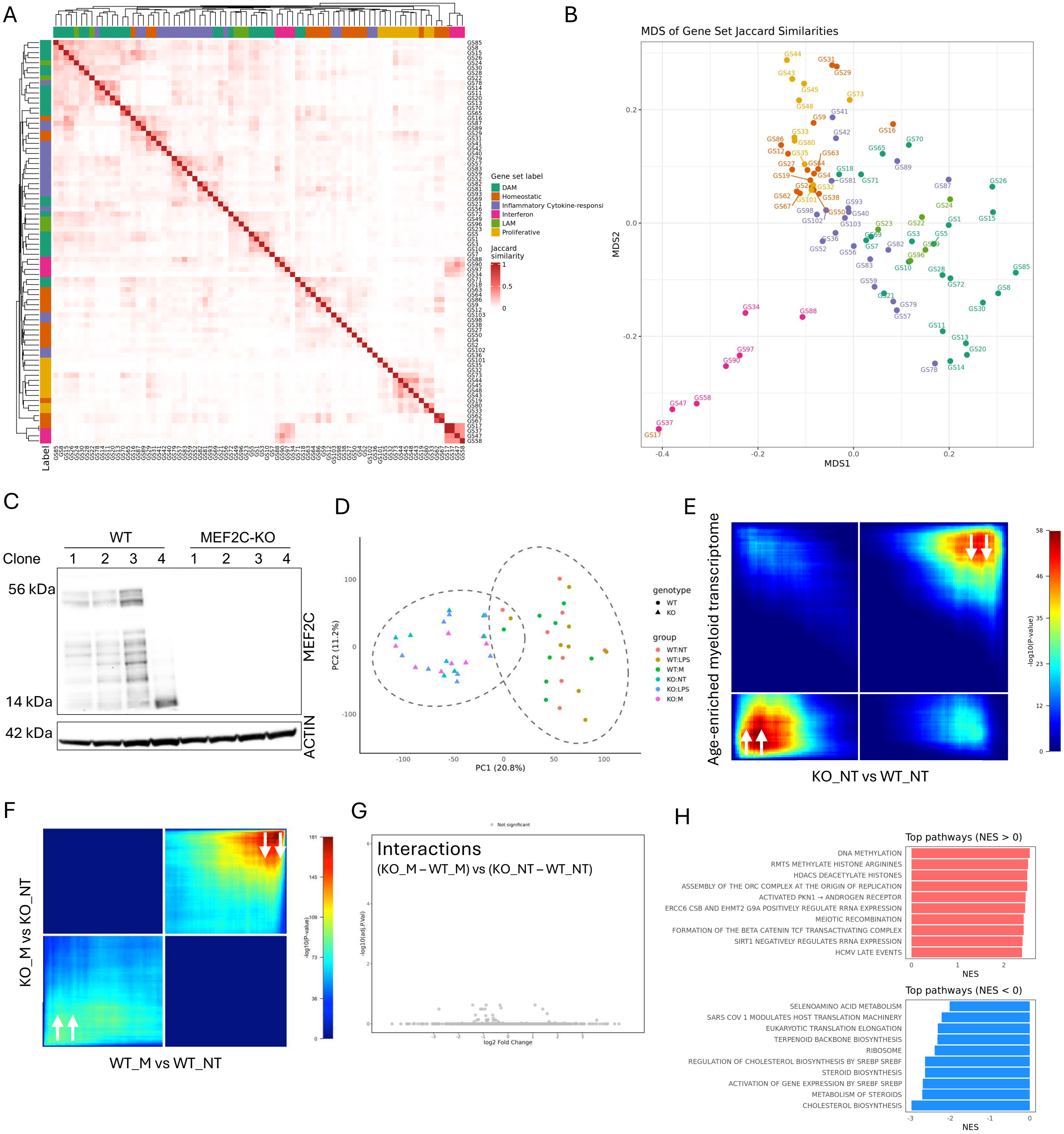

A
Gene set label
DAM
Homeostatic
Inflammatory Cytokine-responsi
Interferon
LAM
Proliferative
Jaccard similarity
1
0.5
0
Label
B
MDS of Gene Set Jaccard Similarities
MDS1
MDS2
C
WT
MEF2C-KO
Clone
1 2 3 4 1 2 3 4
56 kDa
14 kDa
42 kDa
MEF2C
ACTIN
D
PC1 (20.8%)
PC2 (11.2%)
genotype
WT
KO
group
WT:NT
WT:LPS
WT:M
KO:NT
KO:LPS
KO:M
E
Age-enriched myeloid transcriptome
KO_NT vs WT_NT
-log10(P-value)
F
KO_M vs KO_NT
WT_M vs WT_NT
-log10(P-value)
G
Not significant
Interactions
(KO_M – WT_M) vs (KO_NT – WT_NT)
-log10(adj.P.Val)
log2 Fold Change
H
Top pathways (NES > 0)
DNA METHYLATION
RMTS METHYLATE HISTONE ARGININES
HDACS DEACETYLATE HISTONES
ASSEMBLY OF THE ORC COMPLEX AT THE ORIGIN OF REPLICATION
ACTIVATED PKN1 → ANDROGEN RECEPTOR
ERCC6 CSB AND EHMT2 G9A POSITIVELY REGULATE RRNA EXPRESSION
MEIOTIC RECOMBINATION
FORMATION OF THE BETA CATENIN TCF TRANSACTIVATING COMPLEX
SIRT1 NEGATIVELY REGULATES RRNA EXPRESSION
HCMV LATE EVENTS
NES
Top pathways (NES < 0)
SELENOAMINO ACID METABOLISM
SARS COV 1 MODULATES HOST TRANSLATION MACHINERY
EUKARYOTIC TRANSLATION ELONGATION
TERPENOID BACKBONE BIOSYNTHESIS
RIBOSOME
REGULATION OF CHOLESTEROL BIOSYNTHESIS BY SREBP SREBF
STEROID BIOSYNTHESIS
ACTIVATION OF GENE EXPRESSION BY SREBF SREBP
METABOLISM OF STEROIDS
CHOLESTEROL BIOSYNTHESIS
NES

Supplementary Figure 1. Gene set similarity structure and validation of MEF2C perturbation effects in myeloid models

(A) Heatmap of pairwise Jaccard similarities among curated myeloid gene sets (Supplementary Data 1), hierarchically clustered to reveal shared gene content and relationships between disease-associated microglial, homeostatic, inflammatory, interferon-responsive, lipid-associated macrophage, and proliferative cluster marker genes. Colored annotations indicate gene set categories.

(B) Multidimensional scaling (MDS) representation of gene set similarities based on Jaccard distance. Each point represents a gene set, colored by functional category, illustrating segregation and partial overlap between major myeloid transcriptional programs. GS - gene set identifier (Supplementary Data 1)

(C) Immunoblot validation of MEF2C knockout in iPSC-derived microglia. Representative blots show loss of MEF2C protein in MEF2C-KO clones compared to wild-type (WT) controls, with Actin serving as a loading control.

(D) Principal component analysis (PCA) of bulk transcriptomic profiles from WT and MEF2C-KO cells across experimental conditions. Dashed ellipses indicate clustering by genotype.

(E) Rank-rank hypergeometric overlap (RRHO) comparing age-enriched myeloid transcriptomic signatures (from [44], Supplementary Table 14, adj.p.val < 0.05) with MEF2C-KO transcriptome (KO_NT vs WT_NT).

(F) Rank-rank hypergeometric overlap (RRHO) comparing transcriptomes in WT and MEF2C-KO treated with myelin fragments.

In E and F, p-value in color temperature scale represents FDR corrected p-value of hypergeometric overlap test (equivalent to one-sided Fisher's exact test). White arrows indicate whether genes are up- or downregulated.

(G) Volcano plot illustrating interaction analysis comparing differential expression between KO and WT conditions with response to treatment with myelin fragments.

(H) Gene set enrichment analysis of ranked interaction transcriptome revealing changes where KO iMGLs react differently to myelin challenge than WT iMGLs. Bar plots show the top positively (NES > 0) and negatively (NES < 0) enriched. Input lists, and pathway outcomes related to transcriptomic analysis are in Supplementary Data 2.

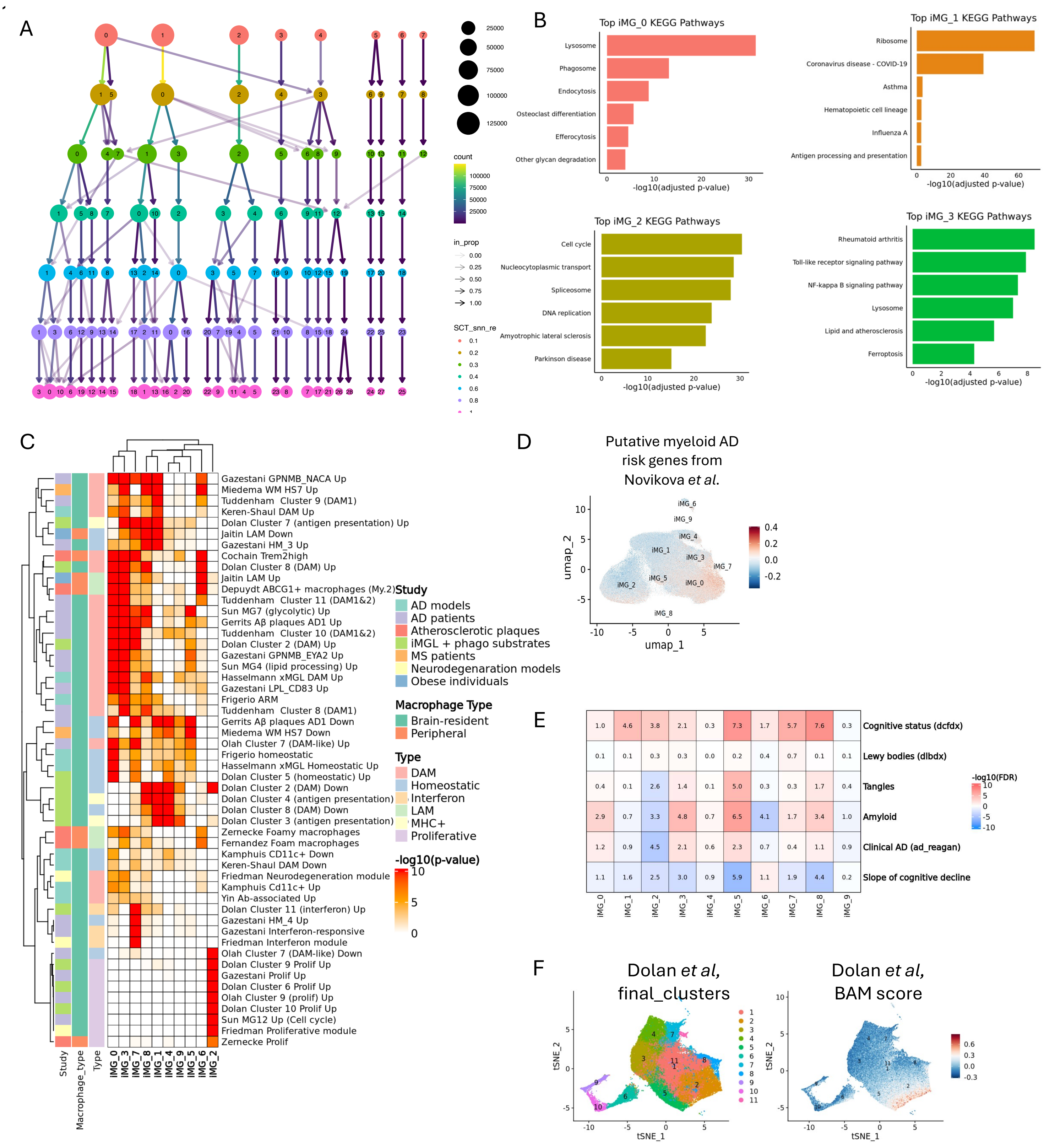

BAM score from Sankowski et al., C19 (Supplementary Data 1)

Supplementary Figure 2. Comparison of iMGLs clusters with published myeloid gene sets and myeloid AD risk genes

(A) The clustree plot was generated using clustree *v0.4.4*. Each tier in the plot corresponds to a distinct clustering resolution. Lines indicate the cell contributions from previous clusters, while the circle size reflects the number of cells in each cluster

(B) KEGG pathway enrichment analysis for selected iMGLs clusters. Bar plots display the top significantly enriched pathways (−log10 adjusted p-values), revealing distinct functional profiles across clusters

(C) Hypergeometric overlap results showing enrichment of myeloid gene sets across iMGLs cluster marker genes (source Supplementary Data 1). Color intensity represents −log10(FDR-adjusted p-value) from hypergeometric testing (Supplementary Data 6).

(D) UMAP feature plots showing the spatial distribution putative myeloid AD risk genes from Novikova et al.[7] across iMG clusters

(E) Correlation of iMGLs clusters with clinical and pathological traits in AD. Enrichment was performed separately for genes positively and negatively correlated with each trait in cluster marker genes (Supplementary Data 1). Coloration of each box relates to the strength and directionality of each association. Red corresponds to the hypergeometric overlap testing the intersection of cluster marker genes and genes upregulated (positively associated) with the trait, while blue corresponds to hypergeometric overlap testing intersection between cluster marker genes and genes downregulated (negatively associated) with the trait. AD traits from [13] Supplementary Table 3.

(F) UMAP representation of iMGLs treated with phagocytic substrates from [43] with main clusters labelled (left) with BAM score module added (right) BAM score from [48] Supplementary Table 3, C19.

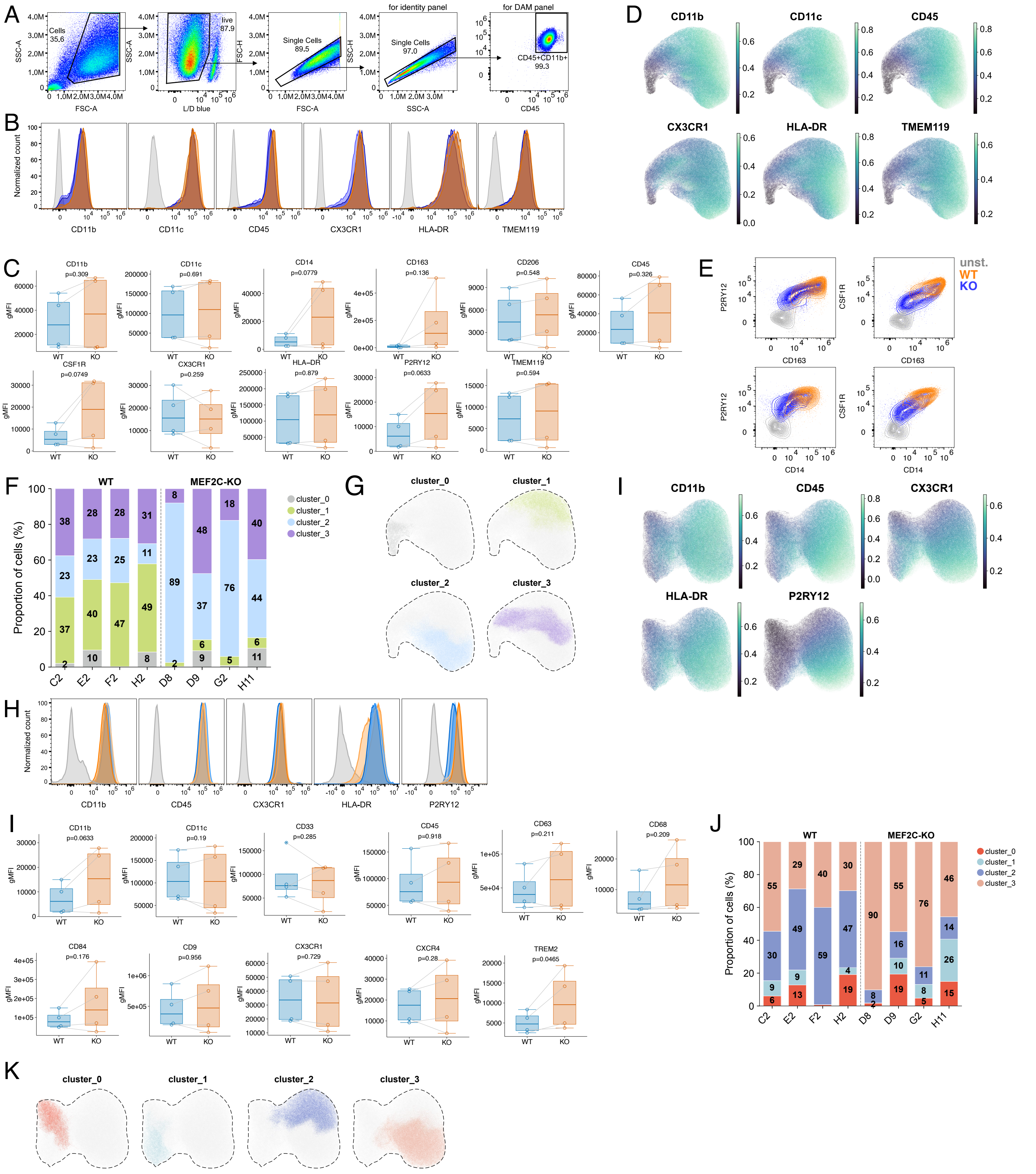
A
Cells 35.6
live 87.9
Single Cells 89.5
for identity panel
Single Cells 97.0
for DAM panel
CD45+CD11b+ 99.3
SSC-A
FSC-A
L/D blue
FSC-H
SSC-H
CD45
B
Normalized count
CD11b
CD11c
CD45
CX3CR1
HLA-DR
TMEM119
C
CD11b p=0.309
CD11c p=0.691
CD14 p=0.0779
CD163 p=0.136
CD206 p=0.548
CD45 p=0.326
CSF1R p=0.0749
CX3CR1 p=0.259
HLA-DR p=0.879
P2RY12 p=0.0633
TMEM119 p=0.594
gMFI
WT
KO
D
CD11b
CD11c
CD45
CX3CR1
HLA-DR
TMEM119
E
unst.
WT
KO
P2RY12
CSF1R
CD163
CD14
F
WT
MEF2C-KO
Proportion of cells (%)
cluster_0
cluster_1
cluster_2
cluster_3
C2
E2
F2
H2
D8
D9
G2
H11
G
cluster_0
cluster_1
cluster_2
cluster_3
I
CD11b
CD45
CX3CR1
HLA-DR
P2RY12
H
Normalized count
CD11b
CD45
CX3CR1
HLA-DR
P2RY12
I
CD11b p=0.0633
CD11c p=0.19
CD33 p=0.285
CD45 p=0.918
CD63 p=0.211
CD68 p=0.209
CD84 p=0.176
CD9 p=0.956
CX3CR1 p=0.729
CXCR4 p=0.28
TREM2 p=0.0465
gMFI
WT
KO
J
WT
MEF2C-KO
Proportion of cells (%)
cluster_0
cluster_1
cluster_2
cluster_3
C2
E2
F2
H2
D8
D9
G2
H11
K
cluster_0
cluster_1
cluster_2
cluster_3

Supplementary Figure 3. High-dimensional flow cytometry reveals expansion of BAM- and DAM-like microglial populations upon MEF2C loss

(A) Gating strategy for high-dimensional flow cytometry analysis of WT and MEF2C-KO iPSC-derived microglia (iMGLs). Cells were gated sequentially to exclude debris and doublets, followed by selection of live cells and $CD45^{+}CD11b^{+}$ myeloid cells used for downstream analyses.

(B) Representative flow cytometry histograms showing expression of myeloid markers. Grey histograms represent unstained controls.

(C) Quantification of geometric mean fluorescence intensity (gMFI) for markers included in the myeloid identity panel in WT and MEF2C-KO iMGLs.

(D) UMAP embedding of single-cell cytometry data generated using CytoVI and colored by normalized expression of the indicated markers.

(E) Contour plots showing co-expression of selected markers highlighting overlapping expression patterns of BAM-associated markers (CD14, CD163) and canonical microglial markers (P2RY12, CSF1R).

(F) Proportion of cells belonging to each CytoVI-defined cluster across individual WT and MEF2C-KO iMGL clones.

(G) Visualization of clusters identified by unsupervised clustering projected onto the CytoVI UMAP embedding.

(H) Representative flow cytometry histograms showing expression of DLAM-associated markers used in the second antibody panel.

(I) Quantification of geometric mean fluorescence intensity (gMFI) for markers included in the DLAM panel in WT and MEF2C-KO iMGLs.

(J) Proportion of cells assigned to each cluster across WT and MEF2C-KO iMGL clones based on clustering of the DLAM panel.

(K) CytoVI UMAP embedding showing spatial distribution of clusters identified using the DLAM marker panel.

For all box plots (C and I), points represent independent clone replicates. Boxes indicate the interquartile range with the center line representing the median. P values were calculated using linear mixed-effects models followed by post hoc pairwise comparisons using estimated marginal means (emmeans); exact P values are shown.

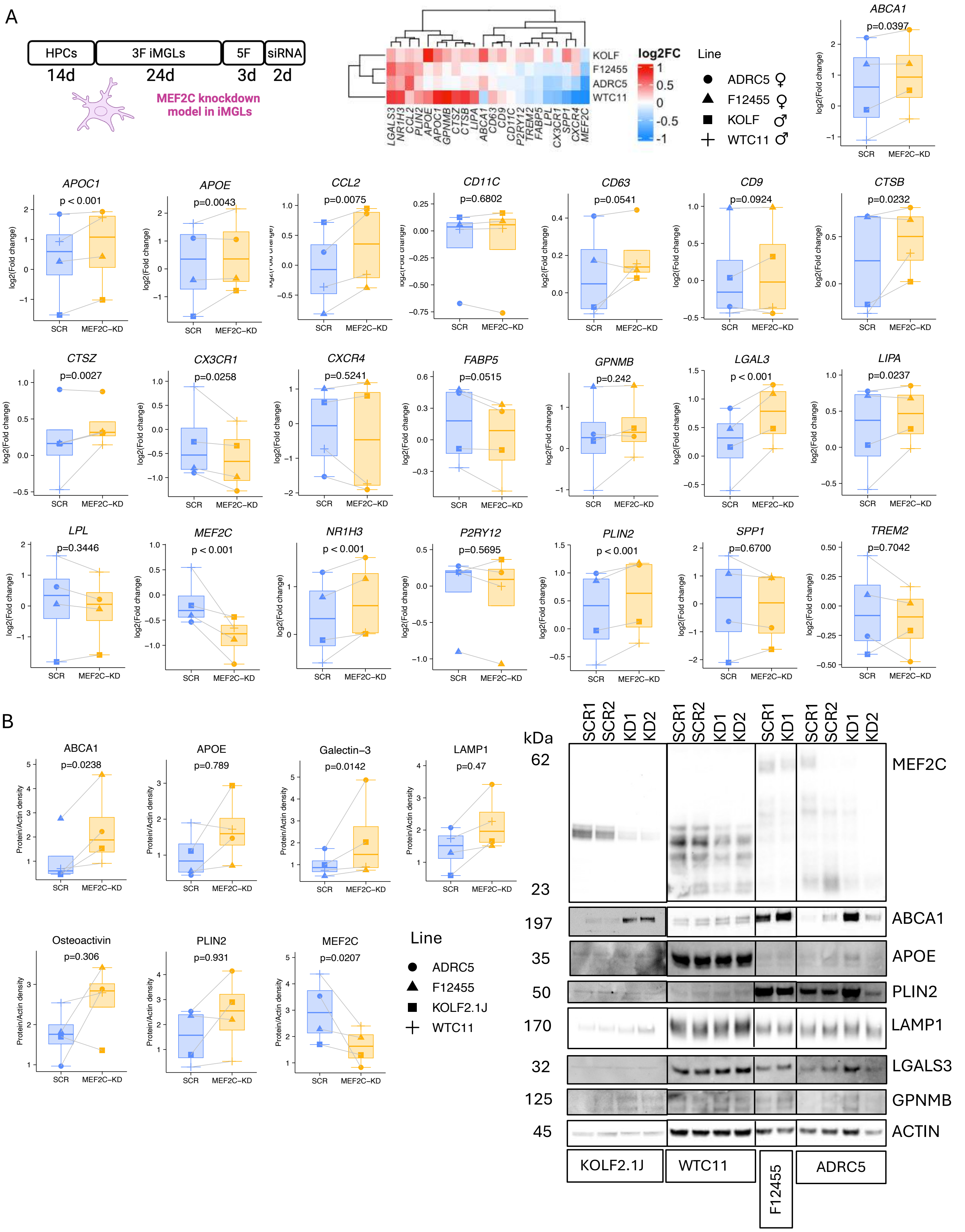
A
HPCs
14d
3F iMGLs
24d
5F
3d
siRNA
2d
MEF2C knockdown model in iMGLs
KOLF
F12455
ADRC5
WTC11
log2FC
Line
ADRC5 ♀
F12455 ♀
KOLF ♂
WTC11 ♂
ABCA1 p=0.0397
APOC1 p < 0.001
APOE p=0.0043
CCL2 p=0.0075
CD11C p=0.6802
CD63 p=0.0541
CD9 p=0.0924
CTSB p=0.0232
CTSZ p=0.0027
CX3CR1 p=0.0258
CXCR4 p=0.5241
FABP5 p=0.0515
GPNMB p=0.242
LGAL3 p < 0.001
LIPA p=0.0237
LPL p=0.3446
MEF2C p < 0.001
NR1H3 p < 0.001
P2RY12 p=0.5695
PLIN2 p < 0.001
SPP1 p=0.6700
TREM2 p=0.7042
log2(Fold change)
SCR
MEF2C–KD
B
ABCA1 p=0.0238
APOE p=0.789
Galectin–3 p=0.0142
LAMP1 p=0.47
Osteoactivin p=0.306
PLIN2 p=0.931
MEF2C p=0.0207
Protein/Actin density
Line
ADRC5
F12455
KOLF2.1J
WTC11
kDa
SCR1 SCR2 KD1 KD2
62
23
197
35
50
170
32
125
45
MEF2C
ABCA1
APOE
PLIN2
LAMP1
LGALS3
GPNMB
ACTIN
KOLF2.1J
WTC11
F12455
ADRC5

Supplementary Figure 4. MEF2C knockdown in human iPSC-derived microglia induces a disease-associated transcriptional and protein signature

(A) Experimental schematic of MEF2C knockdown in iPSC-derived microglia (iMGLs) using siRNA following hematopoietic progenitor cell (HPC) differentiation. Heatmap (top) shows log2 fold changes of selected genes across independent iPSC lines (WTC11, ADRC5, F12455, and KOLF). Each line was compared to its own SCR control only for visualization purposes. Box plots show gene expression changes (log2 fold change) for canonical disease-associated microglial DLAM markers, and MEF2C in scrambled control (SCR) and KD conditions in iMGLs.

(B) Protein-level validation of MEF2C knockdown effects. Left, quantification of protein expression levels in SCR and KD iMGLs across independent lines. Box plots depict protein density over actin density used as a loading control. Right, representative immunoblots. Uncropped western blot images are in Supplementary Figure 8.

Points represent individual iPSC lines; paired measurements are connected. P values were calculated using linear mixed-effects models followed by post hoc pairwise comparisons using estimated marginal means (emmeans); exact p-values are shown.

1

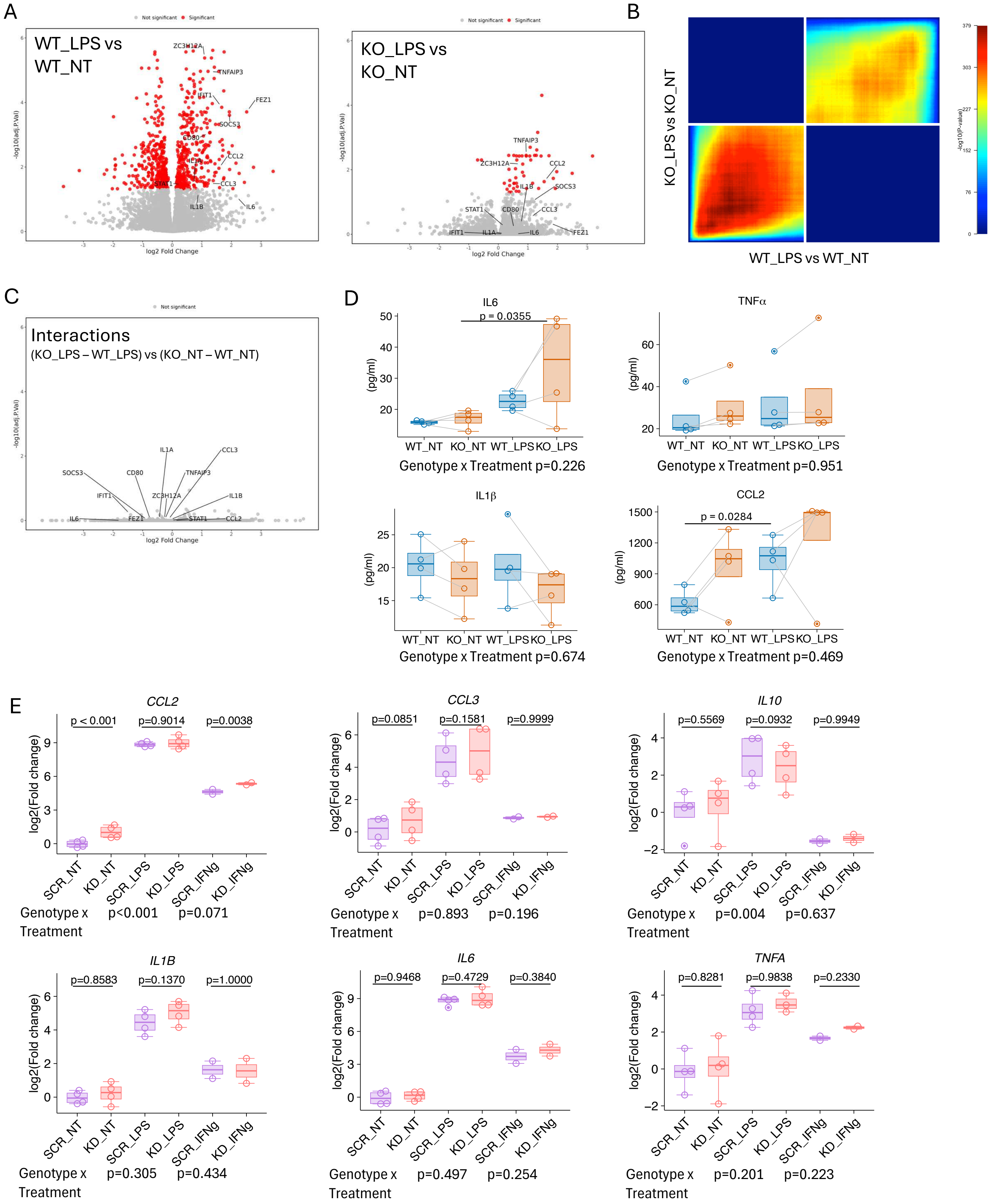
A
Not significant
Significant
WT_LPS vs
WT_NT
-log10(adj.P.Val)
log2 Fold Change
ZC3H12A
TNFAIP3
IFIT1
FEZ1
SOCS3
CD80
IL1A
CCL2
STAT1
CCL3
IL1B
IL6
KO_LPS vs
KO_NT
B
KO_LPS vs KO_NT
WT_LPS vs WT_NT
-log10(P-value)
379
303
227
152
76
0
C
Interactions
(KO_LPS – WT_LPS) vs (KO_NT – WT_NT)
D
IL6
p = 0.0355
(pg/ml)
WT_NT
KO_NT
WT_LPS
KO_LPS
Genotype x Treatment p=0.226
TNFα
Genotype x Treatment p=0.951
IL1β
Genotype x Treatment p=0.674
CCL2
p = 0.0284
Genotype x Treatment p=0.469
E
CCL2
p < 0.001
p=0.9014
p=0.0038
log2(Fold change)
SCR_NT
KD_NT
SCR_LPS
KD_LPS
SCR_IFNg
KD_IFNg
Genotype x
Treatment
p<0.001
p=0.071
CCL3
p=0.0851
p=0.1581
p=0.9999
p=0.893
p=0.196
IL10
p=0.5569
p=0.0932
p=0.9949
p=0.004
p=0.637
IL1B
p=0.8583
p=0.1370
p=1.0000
p=0.305
p=0.434
IL6
p=0.9468
p=0.4729
p=0.3840
p=0.497
p=0.254
TNFA
p=0.8281
p=0.9838
p=0.2330
p=0.201
p=0.223

Supplementary Figure 5. MEF2C loss does not exacerbate inflammatory responses to LPS or IFNγ stimulation

(A) Volcano plots showing differential gene expression in response to LPS stimulation in WT iMGLs (WT_LPS vs WT_NT; left) and KO iMGLs (KO_LPS vs KO_NT; right). Red points denote significantly differentially expressed genes (adj.p < 0.05) (Supplementary Data 2).

(B) Rank–rank hypergeometric overlap (RRHO) analysis comparing transcriptional responses to LPS stimulation in WT and KO iMGLs. Heatmaps depict regions of concordant gene regulation between WT_LPS vs WT_NT and KO_LPS vs KO_NT comparisons.

(C) Volcano plot showing interaction analysis testing whether LPS-induced transcriptional responses differ between genotypes (KO_LPS − WT_LPS versus KO_NT − WT_NT) (Supplementary Data 2)

(D) Cytokine secretion measured by ELISA following LPS stimulation in Wt and KO iMGLs. Box plots show concentrations of IL-6, TNFα, IL-1β, and CCL2 across WT and KO iMGLs under untreated (NT) and LPS-treated conditions. Each dot represents an independent clone.

(E) Transcriptional responses to IFNγ and LPS stimulation assessed by quantitative gene expression analysis (qPCR). Box plots show log2 fold changes for canonical inflammatory genes (*CCL2, CCL3, IL10, IL1B, IL6,* and *TNFA*) across scrambled control (SCR) and MEF2C knockdown (KD) conditions, under NT, LPS, and IFNγ treatments. Each dot represents independent differentiation.

In D and E Linear mixed-effects models were used to assess genotype, treatment, and interaction effects followed by post hoc pairwise comparisons using estimated marginal means (emmeans); exact p-values are shown. Genotype × treatment interaction p-values are indicated below each panel.

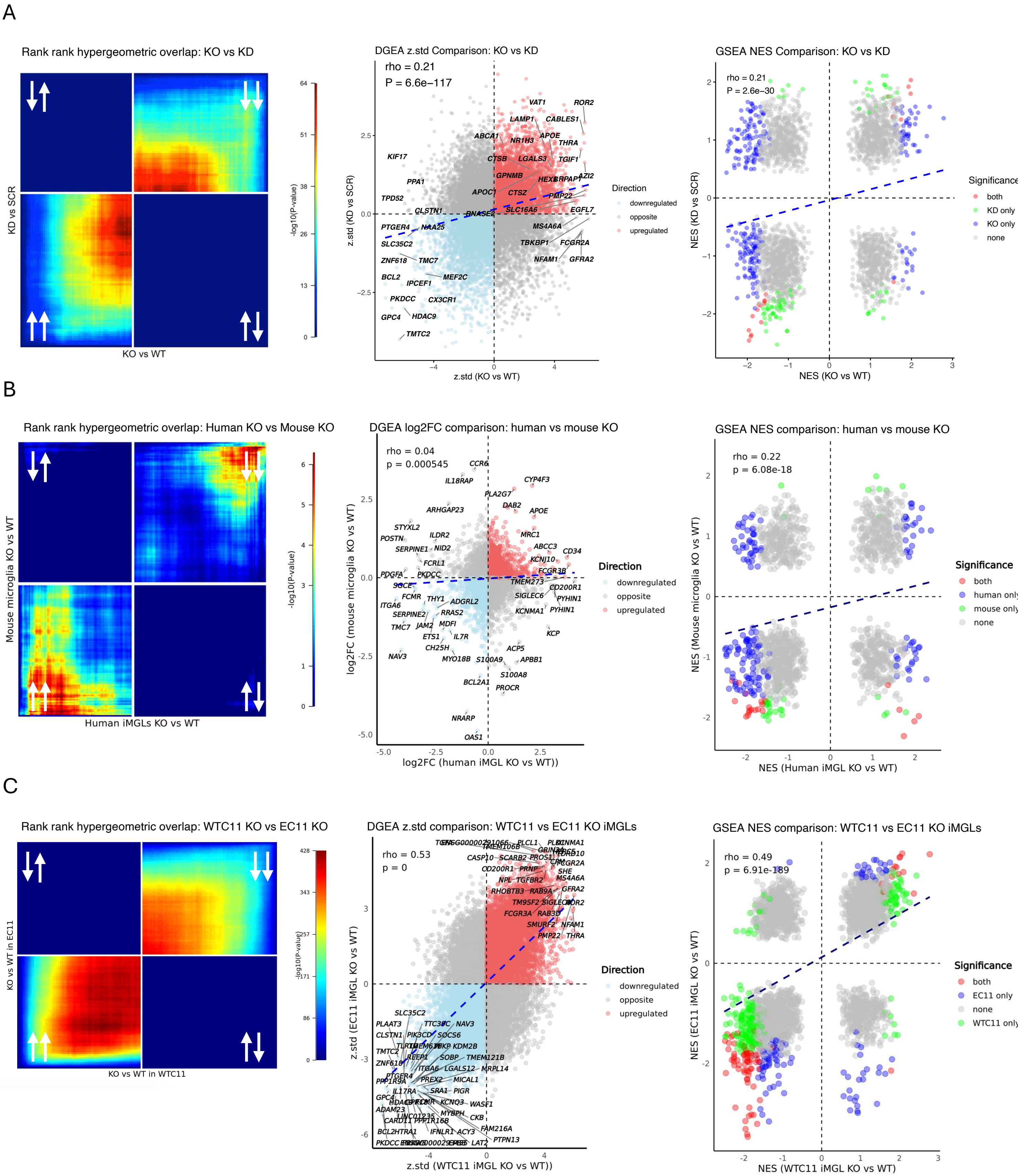
A
Rank rank hypergeometric overlap: KO vs KD
KD vs SCR
KO vs WT
-log10(P-value)
DGEA z.std Comparison: KO vs KD
rho = 0.21
P = 6.6e−117
z.std (KO vs WT)
z.std (KD vs SCR)
Direction
downregulated
opposite
upregulated
GSEA NES Comparison: KO vs KD
rho = 0.21
P = 2.6e−30
NES (KO vs WT)
NES (KD vs SCR)
Significance
both
KD only
KO only
none
B
Rank rank hypergeometric overlap: Human KO vs Mouse KO
Mouse microglia KO vs WT
Human iMGLs KO vs WT
-log10(P-value)
DGEA log2FC comparison: human vs mouse KO
rho = 0.04
p = 0.000545
log2FC (human iMGL KO vs WT))
log2FC (mouse microglia KO vs WT)
Direction
downregulated
opposite
upregulated
GSEA NES comparison: human vs mouse KO
rho = 0.22
p = 6.08e-18
NES (Human iMGL KO vs WT)
NES (Mouse microglia KO vs WT)
Significance
both
human only
mouse only
none
C
Rank rank hypergeometric overlap: WTC11 KO vs EC11 KO
KO vs WT in EC11
KO vs WT in WTC11
-log10(P-value)
DGEA z.std comparison: WTC11 vs EC11 KO iMGLs
rho = 0.53
p = 0
z.std (WTC11 iMGL KO vs WT))
z.std (EC11 iMGL KO vs WT)
Direction
downregulated
opposite
upregulated
GSEA NES comparison: WTC11 vs EC11 KO iMGLs
rho = 0.49
p = 6.91e-189
NES (WTC11 iMGL KO vs WT)
NES (EC11 iMGL KO vs WT)
Significance
both
EC11 only
none
WTC11 only

Supplementary Figure 6. Transcriptional responses to MEF2C perturbation across models, species, and iMGL lines

(A) Comparison of transcriptional changes induced by MEF2C knockout (KO) in iMGLs versus knockdown (KD) in THP-1 macrophages Left, rank–rank hypergeometric overlap (RRHO) analysis. Middle, comparison of gene-level differential expression statistics (z-scores) between KO vs WT and KD vs scrambled control (SCR), with representative concordantly regulated genes highlighted. Right, comparison of gene set enrichment analysis (GSEA) normalized enrichment scores (NES) between KO and KD conditions

(B) Cross-species comparison of MEF2C-dependent transcriptional responses between human iMGLs and mouse microglia. Left, RRHO analysis comparing human iMGL KO vs WT with mouse microglial KO vs WT datasets. Middle, comparison of log2 fold changes for differentially expressed genes between species. Right, concordance of GSEA NES values across human and mouse MEF2C-deficient microglia, highlighting shared pathway-level responses.

(C) Reproducibility of MEF2C knockout transcriptional effects across independent human iMGL lines (WTC11 and EC11(raw data: [31], reprocessed data: Supplementary Data 2)). Left, RRHO analysis comparing KO vs WT signatures between lines. Middle, comparison of differential expression z-scores across lines with concordantly regulated genes highlighted. Right, correlation of GSEA NES values between WTC11 and EC11 KO iMGLs, demonstrating consistent pathway-level effects.

Across panels, dashed lines indicate correlation trends; colors denote concordant regulation in both datasets, regulation unique to one dataset, or non-significant changes, as indicated in the legends. Correlation coefficients (Spearman's ρ) and p-values are shown.

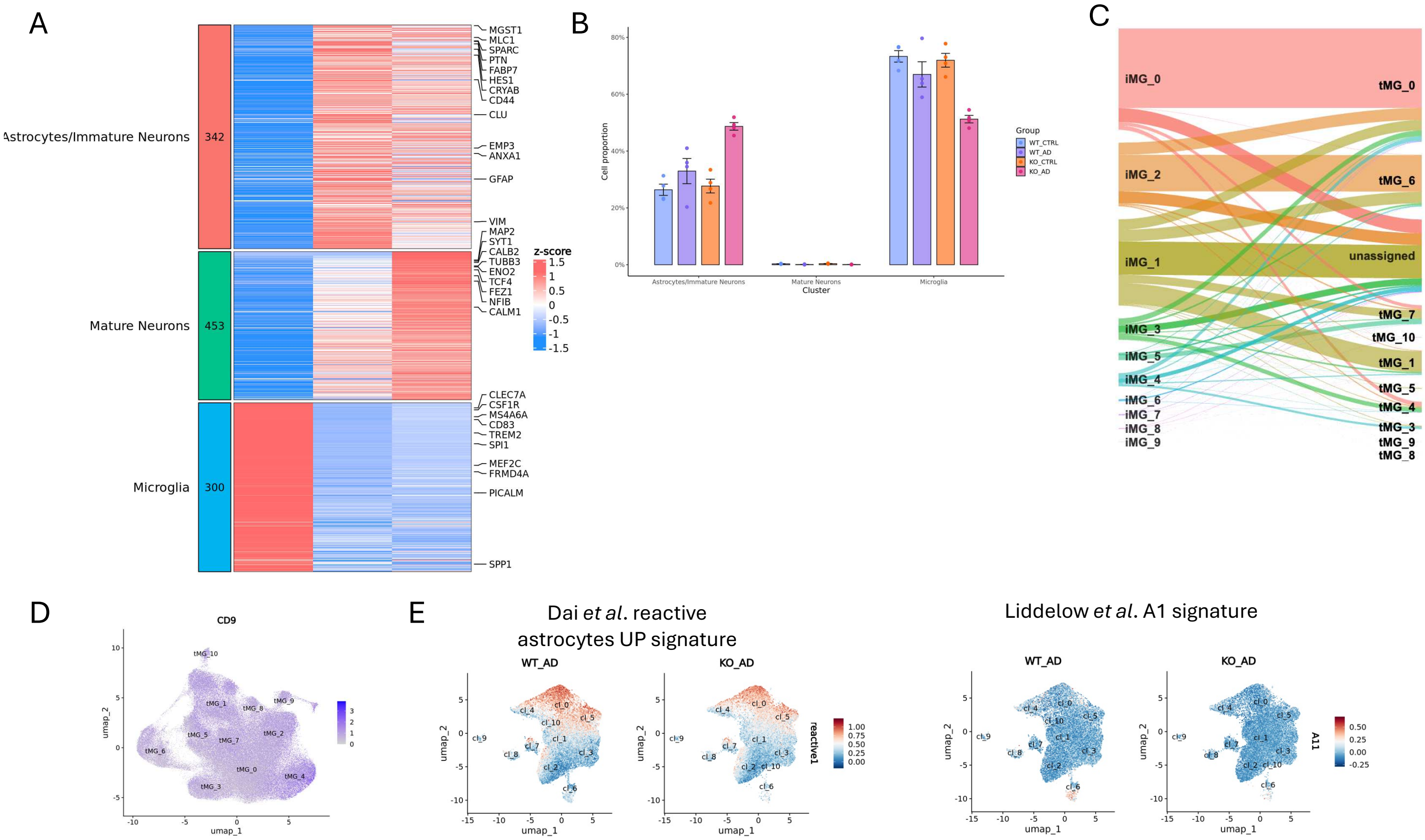

A
Astrocytes/Immature Neurons
342
Mature Neurons
453
Microglia
300
MGST1
MLC1
SPARC
PTN
FABP7
HES1
CRYAB
CD44
CLU
EMP3
ANXA1
GFAP
VIM
MAP2
SYT1
CALB2
TUBB3
ENO2
TCF4
FEZ1
NFIB
CALM1
CLEC7A
CSF1R
MS4A6A
CD83
TREM2
SPI1
MEF2C
FRMD4A
PICALM
SPP1
z-score
1.5
1
0.5
0
-0.5
-1
-1.5
B
Cell proportion
80%
60%
40%
20%
0%
Astrocytes/Immature Neurons
Mature Neurons
Microglia
Cluster
Group
WT_CTRL
WT_AD
KO_CTRL
KO_AD
C
iMG_0
iMG_2
iMG_1
iMG_3
iMG_5
iMG_4
iMG_6
iMG_7
iMG_8
iMG_9
tMG_0
tMG_6
unassigned
tMG_7
tMG_10
tMG_1
tMG_5
tMG_4
tMG_3
tMG_9
tMG_8
D
CD9
umap_2
umap_1
tMG_10
tMG_1
tMG_8
tMG_9
tMG_5
tMG_7
tMG_2
tMG_6
tMG_0
tMG_4
tMG_3
E
Dai *et al*. reactive astrocytes UP signature
Liddelow *et al*. A1 signature
WT_AD
KO_AD
WT_AD
KO_AD
reactive1
A11
umap_2
umap_1

Supplementary Figure 7. Neuron–astrocyte transcriptional states in co-culture with WT and MEF2C-deficient iMGLs.

(A) Heatmap of scaled gene expression (z-score) across major cell classes identified in neuron–astrocyte–microglia co-cultures, highlighting astrocytes/immature neurons, mature neurons, and microglia. Selected marker genes are shown on the right.

(B) Quantification of cell-type proportions across experimental groups (WT_CTRL, WT_AD, KO_CTRL, KO_AD). Bars represent mean ± s.e.m.

(C) Sankey diagram illustrating transitions between monoculture microglial clusters (iMG) and triculture microglial clusters (tMG)

(D) Feature plot showing expression of the DLAM marker CD9 across tMG clusters.

(E) UMAP feature plots showing enrichment of published reactive astrocyte gene signatures, including human reactive astrocytes signature and mouse reactive signature (A1) in AD neurons/astrocytes subset cocultured with WT and =KO iMGLs.

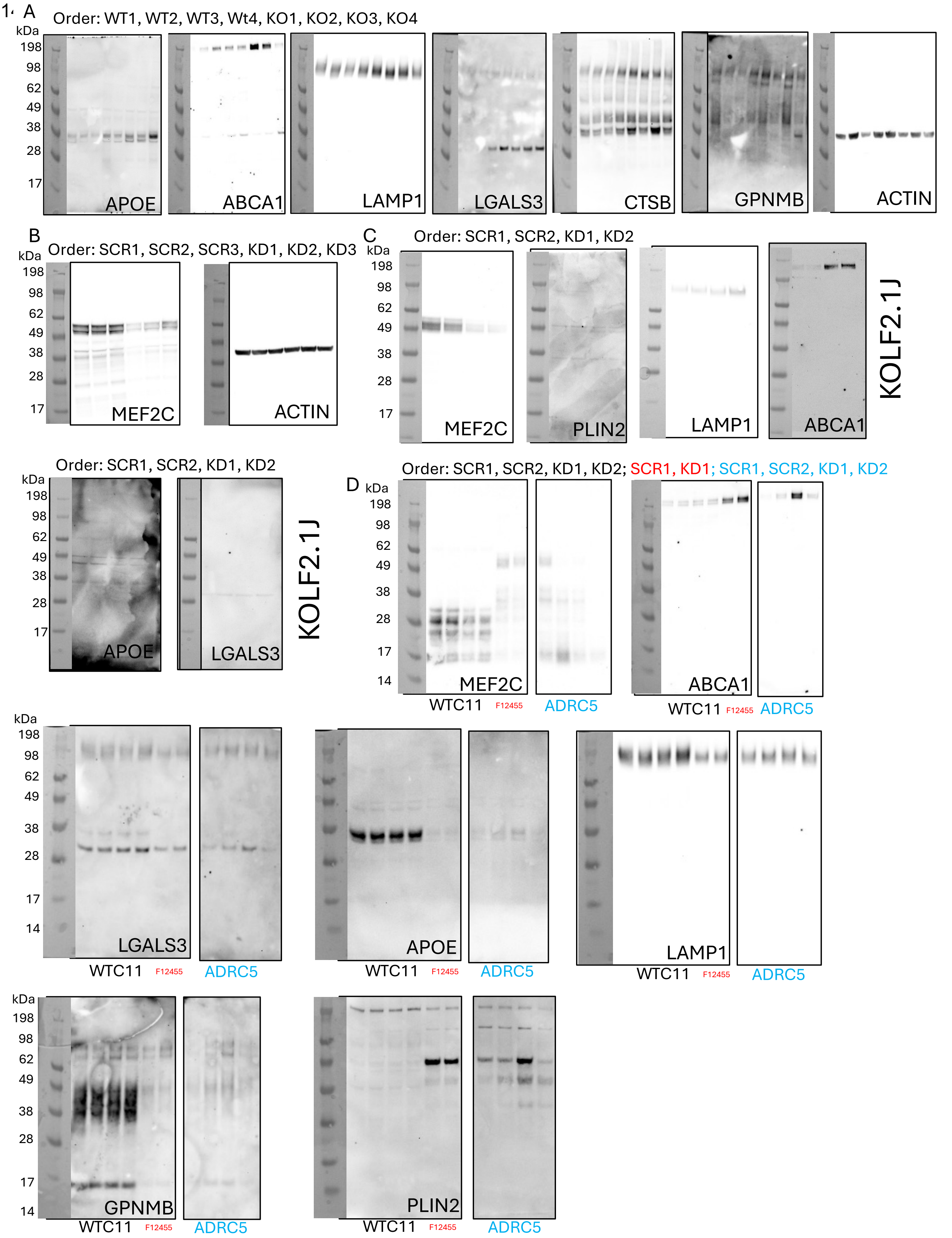
A
Order: WT1, WT2, WT3, Wt4, KO1, KO2, KO3, KO4
kDa
198
98
62
49
38
28
17
APOE
ABCA1
LAMP1
LGALS3
CTSB
GPNMB
ACTIN
B
Order: SCR1, SCR2, SCR3, KD1, KD2, KD3
MEF2C
ACTIN
C
Order: SCR1, SCR2, KD1, KD2
MEF2C
PLIN2
LAMP1
ABCA1
KOLF2.1J
Order: SCR1, SCR2, KD1, KD2
APOE
LGALS3
KOLF2.1J
D
Order: SCR1, SCR2, KD1, KD2; SCR1, KD1; SCR1, SCR2, KD1, KD2
14
MEF2C
ABCA1
WTC11
F12455
ADRC5
LGALS3
APOE
LAMP1
GPNMB
PLIN2

Supplementary Figure 8. Uncropped western blot images

(A) Uncropped WB images for Figure 5L (iMGL with complete genetic inactivation of MEF2C)

(B) Uncropped WB images for Figure 7A (THP-1 macrophages with partial reduction of MEF2C)

(C) Uncropped WB images for Supplementary Figure 6B (iMGL with partial reduction of MEF2C)
